# Paediatric outpatient antibiotic utilization patterns and use of healthcare services before, during and after the COVID-19 pandemic: interrupted time series analysis using data from Norway and Japan

**DOI:** 10.1093/jac/dkag217

**Published:** 2026-06-18

**Authors:** Nhung T H Trinh, Toshiki Fukasawa, Takanori Yanai, Takayuki Okura, Atsushi Takayama, Takamasa Sakai, Olaug M Reiakvam, Hedvig M E Nordeng, Koji Kawakami

**Affiliations:** Pharmacoepidemiology and Drug Safety Research Group, Department of Pharmacy, Faculty of Mathematics and Natural Sciences, University of Oslo, Oslo 0316, Norway; Department of Pharmacoepidemiology, Graduate School of Medicine and Public Health, Kyoto University, Kyoto, Japan; Department of Pharmacoepidemiology, Graduate School of Medicine and Public Health, Kyoto University, Kyoto, Japan; Department of Pharmacoepidemiology, Graduate School of Medicine and Public Health, Kyoto University, Kyoto, Japan; Department of Pharmacoepidemiology, Graduate School of Medicine and Public Health, Kyoto University, Kyoto, Japan; Pharmacoepidemiology and Drug Safety Research Group, Department of Pharmacy, Faculty of Mathematics and Natural Sciences, University of Oslo, Oslo 0316, Norway; Drug Informatics, Faculty of Pharmacy, Meijo University, Nagoya, Aichi, Japan; Department of Microbiology, Oslo University Hospital, Rikshospitalet, Oslo, Norway; Pharmacoepidemiology and Drug Safety Research Group, Department of Pharmacy, Faculty of Mathematics and Natural Sciences, University of Oslo, Oslo 0316, Norway; Department of Child Health and Development, Norwegian Institute of Public Health, Oslo, Norway; Department of Pharmacoepidemiology, Graduate School of Medicine and Public Health, Kyoto University, Kyoto, Japan

## Abstract

**Objectives:**

Quantification of prescription of antimicrobial agents and use of paediatric outpatient services before, during and after the COVID-19 pandemic.

**Methods:**

We conducted a population-based study using Norwegian linked health registries and Japanese claims (2018–2023). Paediatric antibiotic prescription rates, broad-spectrum use, and proportion of antibiotic prescriptions with prior presumed bacterial infection diagnoses were analysed monthly, overall and by age groups and sex. Interrupted time series analyses were performed to evaluate pandemic-related changes, expressed in rate ratio (RR) and its CI, using March 2020 as the interruption point and the pre-pandemic trend/level as reference.

**Results:**

Data on 5.5 million children and 19.5 million antibiotic prescriptions were analysed. Before the pandemic, antibiotic prescribing was higher in Japan (120–200/1000 children/month) than in Norway (10–20/1000). At pandemic onset, rates fell by 45% in Norway (RR = 0.55; 95% CI, 0.45–0.67) and by 53% in Japan (RR = 0.47; 95% CI, 0.41–0.55), then by 2023 had returned to expected levels. Broad-spectrum antibiotic use was much higher in Japan (70%) compared with Norway (10%) before the pandemic. However, Norway experienced a sharp 20% increase whereas Japan remained largely unchanged post-pandemic. The proportion of prescriptions with a prior presumed bacterial diagnosis was between 50% and 65% before the pandemic then decreased modestly by 5%–10% at pandemic onset, followed by gradual rebound in both countries.

**Conclusions:**

The COVID-19 pandemic significantly altered paediatric antibiotic prescribing in both countries. Sustained antibiotic stewardship efforts are needed to ensure appropriate paediatric antibiotic use in the post-pandemic era.

## Introduction

Children are at high risk of being exposed to unnecessary and inappropriate antibiotics, often due to the high incidence of viral respiratory infections in this age group, diagnostic uncertainty and parental expectations. Recent studies from the USA have shown that about one-third of paediatric antibiotic prescriptions may be unnecessary, mostly for viral respiratory tract infections (RTIs).^1^ Corresponding data from other countries, including Norway and Japan, are currently lacking.

Existing literature suggests that the COVID-19 pandemic has had a profound impact on several aspects of healthcare practice worldwide. Several European and Asian countries reported substantial reductions in antibiotic use during the early pandemic period.^2,3^ However, less is known about these trends in the post-pandemic era. Moreover, the impact of the pandemic on prescribing quality has received relatively little attention.^4^

Despite the communicable nature of infectious diseases, few studies have compared antibiotic prescribing patterns across countries and continents. Norway is among the countries with the lowest antibiotic consumption in Europe due to conservative antibiotic prescribing norms.^5^ However, concerns about the quality of prescribing, including the excessive use of broad-spectrum antibiotics, exist in relation to children.^6^ In contrast, Japan has historically reported higher outpatient antibiotic prescription rates both in children and adults, but strong stewardship policies are currently implemented.^7,8^ Both countries implemented similar infection control measures during the pandemic, offering a natural experiment to examine how the pandemic impacted antibiotic use in different settings and how prescribing cultures shape resilience to and recovery from pandemic-related disruptions in antibiotic use. This comparison serves as a mutual learning opportunity not only for Japan and Norway, but also for countries that share similar prescribing cultures yet lack the data needed to investigate comparable questions.

### Objectives

The objectives of this study were 2-fold:

1. Quantification and comparison of the use of antimicrobial agents in paediatric outpatients in Norway and Japan focusing on:
  1. Antibiotic prescription rate
  2. Broad-spectrum antibiotic prescription rate
  3. Proportion of prescription fills/claims that included broad-spectrum antibiotics
  4. Proportion of antibiotic prescription fills/claims with a recorded diagnosis of presumed bacterial infection.
2. Quantification and comparison of the use of paediatric outpatient services in Norway and Japan focusing on:
  1. Paediatric visit rate
  2. Rate of visits associated with a presumed bacterial infection diagnosis.

In Japan, outpatient refers to consultations not resulting in inpatient admission, including visits to office-based clinics and hospital outpatient departments. In Norway, outpatient refers to consultations with GPs and specialists in primary care.

## Methods

### Data sources

We conducted a retrospective observational study utilizing nationwide linked health registries data from Norway and claims data in Japan (January 2018 to December 2023) following recommendations adapted from the STROBE guideline for interrupted time series analysis (Appendix S1, available as Supplementary data at *JAC* Online).^9^

In Norway, data include information on all Norwegian residents (Statistics Norway), dispensed prescriptions at retail pharmacies as recorded in the Norwegian Prescribed Drug Registry (LMR), and diagnostic codes from public and private healthcare providers in primary care recorded in the Norwegian Control and Payment of Health Reimbursements database (KUHR).^10^ In Japan, data were sourced from JMDC Inc., a large administrative database covering claims submitted by health insurance societies across the country, which consists primarily of those who are employees of large businesses, and their dependants.^11^ Both data sources include comprehensive patient-level information, including age, sex, dates of prescription fills or claims, and diagnostic codes.

### Study cohorts

We included all children under 18 years of age living in Norway and Japan during the study period. These children participated in 72 cohorts for each month between January 2018 and December 2023 based on their date of birth and observation period (i.e. death date or end of data availability for Norway; start and end of insurance coverage for Japan). We further categorized them into age groups (0–1, 2–5, 6–9, 10–14, 15–17 years) and sex (male and female).

### Definition of the COVID-19 pandemic

In Norway, the COVID-19 pandemic is considered as the period between February 2020 and May 2023 with the implementation of various mitigation measures.^12^ Key timepoints include March 2020 (declaration of the pandemic), December 2020 (vaccination rollout for adults), September 2021 (vaccination recommendation for adolescents), and January 2022 (vaccination recommendation for young children). The COVID-19 pandemic period in Japan is best defined as the period from 1 April 2020 to 7 May 2023, during which COVID-19 was designated as a class 2 infectious disease.^13^ After 8 May 2023, COVID-19 was classified as a category 5 infectious disease (the same category as influenza and others), and the COVID-19 response in clinics and hospitals was considerably eased. In this study, we used pandemic onset as a single interruption point, given the limited number of observations after May 2023, to provide a stable estimate of overall effects.

### Antibiotic prescription fills and claims

Antibiotics were identified using the Anatomical Therapeutic Chemical (ATC) classification system (J01—Antibacterials for systemic use). Broad-spectrum antibiotics include combinations of penicillins with β-lactamase inhibitors (ATC J01CR), second- and third-generation cephalosporins (J01DC and J01DD), macrolides except erythromycin (J01F except J01FA01) and fluoroquinolones (J01MA) as per definition of the European Surveillance of Antimicrobial Consumption Network.^14^

### Outcome measures

Three primary outcome measures were assessed on a monthly basis: (i) antibiotic prescription rate defined as number of antibiotic prescription fills/claims per 1000 children; (ii) proportion of fills/claims that included broad-spectrum antibiotics among all antibiotic prescription fills/claims; (iii) broad-spectrum antibiotic prescription rate; and (iv) proportion of antibiotic prescription fills/claims with a recorded diagnosis of presumed bacterial infection within 7 days prior to and including the dispensing/claim date. The list of presumed bacterial infections and associated codes in the ICD-10 and the International Classification of Primary Care, 2nd edition (ICPC-2/ICPC-2B) is provided in Table S1.^15^

To better understand healthcare-seeking behaviour and the burden of bacterial diseases, we also included two secondary outcomes: (i) the rate of paediatric visits (per 1000 children per month) and (ii) the rate of visits associated with a presumed bacterial infection diagnosis (per 1000 children per month). Paediatric visits recorded in KUHR and JMDC were counted as distinct encounters on different days, irrespective of visit purpose. A visit was considered infection-related if at least one bacterial infection diagnosis was recorded during that encounter.

### Statistical analysis

We applied interrupted time series (ITS) analysis to evaluate changes in outcome measures, using March 2020, the onset of the COVID-19 pandemic, as the interruption point, yielding 26 timepoints before and 45 timepoints after the interruption.^16^ We fitted a quasi-Poisson regression model with four Fourier terms (i.e. two harmonics) accounting for overdispersion and seasonal components, and a full impact model to estimate (i) the level change and (ii) slope changes expressed in rate ratios (RRs) compared with pre-pandemic level/slope and their 95% CIs. Residual autocorrelation was evaluated using autocorrelation function plots, and when autocorrelation was present (mostly at lag 1), robust standard errors were estimated using the Newey–West method. Model fit and the presence of outliers were examined through residual diagnostic plots.

In addition, we plotted fitted values and predicted values had the pandemic not occurred to facilitate the interpretation of the results via visual inspection. The predicted values were projected based on the pre-pandemic levels, trends and seasonal variations. Analyses were conducted separately for each country, overall, and by age groups and sex. Analyses employed two-sided hypothesis tests with α = 0.05.

### Additional analyses

First, we considered an extended look-back time of 14 days to examine accompanied diagnoses to account for the potential ‘wait-and-see’ prescribing practice. Second, because most GPs in Norway used ICPC-2/ICPC-2B to record the diagnoses, we tested a relaxed definition of presumed bacterial infection considering R74 Upper respiratory infection acute and A78 Infectious disease other/NOS in addition as they are among the most used codes prior to an antibiotic prescription in children.

We used Stata 18 for data management and R/RStudio with the *its2es* package for data analysis and visualization.^16^

## Results

We included data on 1 699 377 children in Norway and 3 844 199 children in Japan from January 2018 through December 2023. During this period, 1 235 424 antibiotic prescriptions were filled in Norway in 551 383 children (32.4%). In Japan, 18 288 406 antibiotic claims were recorded in 2 598 699 (55.8%). Table 1 lists the antibiotics that account for 90% of paediatric antibiotic dispensations in each country. In Norway, 10 different antibiotics made up this share, with only clindamycin classified as broad-spectrum, representing 4% of dispensations. In contrast, Japan had 22 such antibiotics with half of them being broad-spectrum.

**Table 1.** Antibiotics that contributed to 90% of antibiotic dispensations in children during the study period (2018–2023)

ATC	Active substance(s)	Number of dispensations (%)	Number of users (%)
**Norway**			
J01CE02	phenoxymethylpenicillin	529 906 (42.9)	340 424 (20.0)
J01FA01	erythromycin	94 674 (7.7)	70 116 (4.1)
J01CA04	pivampicillin	92 015 (7.4)	65 448 (3.9)
J01CF01	dicloxacillin	70 055 (5.7)	53 745 (3.2)
J01AA04	lymecycline	62 732 (5.1)	35 545 (2.1)
J01CA08	pivmecillinam	59 425 (4.8)	42 182 (2.5)
J01EA01	trimethoprim	58 047 (4.7)	36 657 (2.2)
J01EE01	sulfamethoxazole and trimethoprim	54 834 (4.4)	30 486 (1.8)
**J01FF01**	**clindamycin**	**49 173 (4.0)**	**34 013 (2.0)**
J01AA02	doxycycline	37 233 (3.0)	25 652 (1.5)
**Japan**			
**J01FA09**	**clarithromycin**	**3 483 868 (19.0)**	**1 154 894 (30.0)**
J01CA04	amoxicillin	2 431 161 (13.3)	925 804 (24.1)
**J01DD16**	**cefditoren**	**2 112 408 (11.6)**	**912 166 (23.7)**
**J01DD17**	**cefcapene**	**1 554 970 (8.5)**	**780 840 (20.3)**
**J01DD15**	**cefdinir**	**999 508 (5.5)**	**521 409 (13.6)**
**J01MA22**	**tosufloxacin**	920 827 (5.0)	376 312 (9.8)
**J01CR02**	**amoxicillin and β-lactamase inhibitor**	**496 783 (2.7)**	**232 734 (6.1)**
J01GB11	isepamicin	475 941 (2.6)	77 090 (2.0)
J01XX01	fosfomycin	447 346 (2.4)	197 440 (5.1)
**J01FA10**	**azithromycin**	**424 402 (2.3)**	**264 336 (6.9)**
J01GB09	dibekacin	405 047 (2.2)	75 983 (2.0)
**J01DD13**	**cefpodoxime**	**352 596 (1.9)**	**197 217 (5.1)**
J01FA01	erythromycin	314 497 (1.7)	89 478 (2.3)
J01GB06	amikacin	279 411 (1.5)	41 944 (1.1)
J01AA08	minocycline	256 646 (1.4)	104 376 (2.7)
**J01DC04**	**cefaclor**	**249 721 (1.4)**	**156 252 (4.1)**
J01DB04	cefazolin	238 236 (1.3)	87 906 (2.3)
**J01DD18**	**cefteram**	**231 996 (1.3)**	**119 679 (3.1)**
**J01FA06**	**roxithromycin**	**227 002 (1.2)**	**90 475 (2.4)**
J01CA01	ampicillin	222 253 (1.2)	50 917 (1.3)
J01EE01	sulfamethoxazole and trimethoprim	195 288 (1.1)	9842 (0.3)
J01DH06	tebipenem pivoxil	182 054 (1.0)	86 986 (2.3)

Broad-spectrum antibiotics are highlighted in bold.

### Main outcomes

#### Antibiotic prescription rates

**Figure 1.**
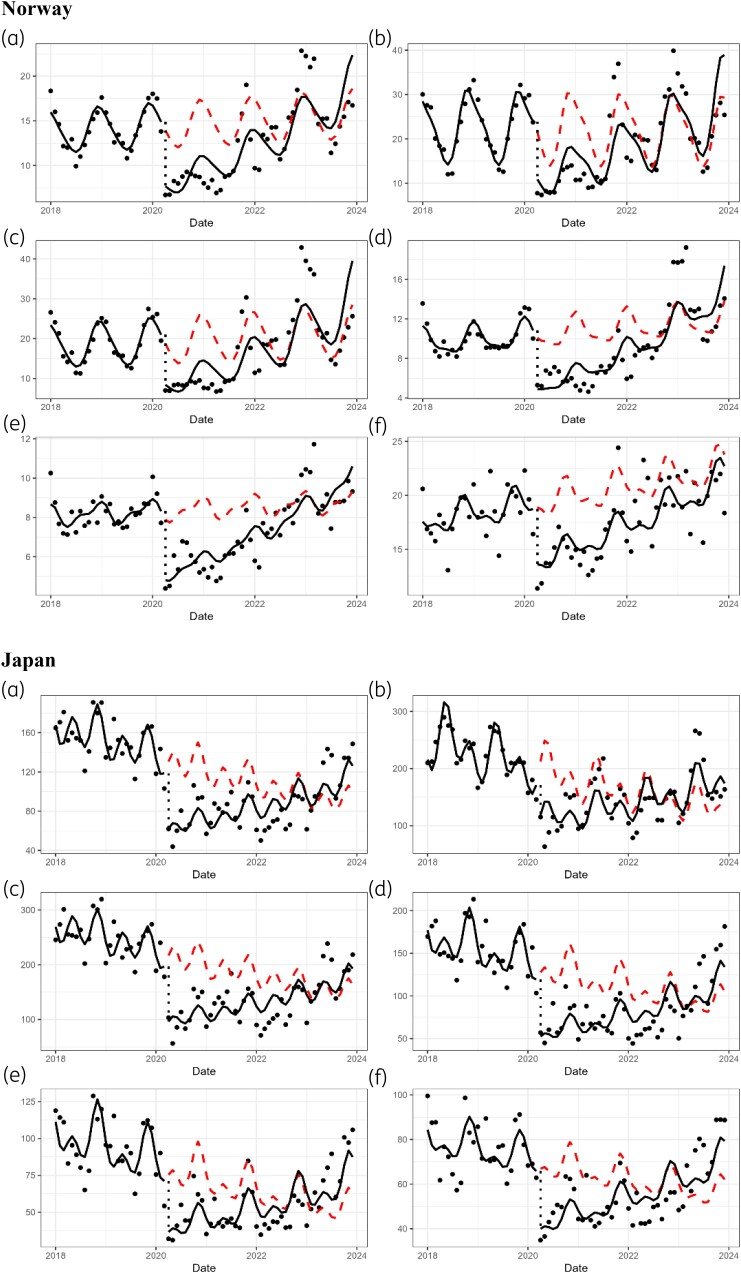
Antibiotic prescription rate overall and by age groups in Norway (top) and Japan (bottom) (solid line: fitted values; dotted line: predicted values had the pandemic not occurred): number of antibiotic prescription fills/claims per 1000 children per month. (a) Overall. (b) Children 0–1 years old. (c) Children 2–5 years old. (d) Children 6–9 years old. (e) Children 10–14 years old. (f) Adolescents 15–17 years old.

Before March 2020, Japan had a substantially higher antibiotic prescription rate (120–200 prescriptions/1000 children/month) than Norway (10–20/1000) but the rates in both countries remained relatively stable (Table 2). The highest rates in both countries were observed among children aged 0–1 and 2–5 years (Figure 1).

**Table 2.** Impact of the pandemic on outcomes of interest (rate ratio with 95% CI), using March 2020 as the interruption point and pre-pandemic trend/level as reference

	Pre-pandemic trend	Change in level	Change in slope
**Norway**	Antibiotic prescription rate per 1000 children		
Overall	1.00 (1.00–1.01)	**0.55 (0.45–0.67)**	**1.02 (1.01–1.03)**
Male children	1.00 (1.00–1.01)	**0.59 (0.50–0.69)**	**1.02 (1.01–1.02)**
Female children	1.00 (1.00–1.01)	**0.51 (0.41–0.64)**	**1.02 (1.01–1.03)**
Children 0–1 years	1.00 (0.99–1.00)	**0.51 (0.39–0.66)**	**1.02 (1.01–1.03)**
Children 2–5 years	1.00 (1.00–1.01)	**0.45 (0.32–0.63)**	**1.03 (1.01–1.04)**
Children 6–9 years	1.00 (1.00–1.01)	**0.49 (0.40–0.60)**	**1.02 (1.01–1.03)**
Adolescents 10–14 years	1.00 (1.00–1.01)	**0.61 (0.54–0.69)**	**1.01 (1.01–1.02)**
Adolescents 15–17 years	1.00 (1.00–1.01)	**0.72 (0.64–0.81)**	**1.01 (1.00–1.01)**
**Japan**	Antibiotic prescription rate per 1000 children		
Overall	0.99 (0.99–1.00)	**0.47 (0.41–0.55)**	**1.02 (1.02–1.03)**
Male children	0.99 (0.99–1.00)	**0.48 (0.42–0.56)**	**1.02 (1.02–1.03)**
Female children	0.99 (0.99–1.00)	**0.46 (0.40–0.54)**	**1.02 (1.02–1.03)**
Children 0–1 years	0.99 (0.99–0.99)	**0.56 (0.46–0.68)**	**1.02 (1.01–1.03)**
Children 2–5 years	0.99 (0.99–1.00)	**0.44 (0.37–0.53)**	**1.02 (1.02–1.03)**
Children 6–9 years	0.99 (0.98–1.00)	**0.41 (0.33–0.51)**	**1.03 (1.02–1.04)**
Adolescents 10–14 years	0.99 (0.98–1.00)	**0.49 (0.40–0.59)**	**1.02 (1.02–1.03)**
Adolescents 15–17 years	0.99 (0.99–1.00)	**0.60 (0.52–0.69)**	**1.02 (1.01–1.03)**
**Norway**	Broad-spectrum antibiotic prescription rate per 1000 children		
Overall	**1.01 (1.00–1.01)**	**0.66 (0.58–0.75)**	**1.01 (1.01–1.02)**
Male children	**1.01 (1.00–1.01)**	**0.68 (0.60–0.77)**	**1.01 (1.00–1.02)**
Female children	**1.00 (1.00–1.01)**	**0.64 (0.54–0.76)**	**1.02 (1.01–1.03)**
Children 0–1 years	**1.02 (1.01–1.02)**	**0.74 (0.61–0.90)**	**1.01 (1.00–1.01)**
Children 2–5 years	**1.01 (1.00–1.02)**	**0.65 (0.53–0.80)**	**1.02 (1.00–1.03)**
Children 6–9 years	**1.01 (1.00–1.01)**	**0.67 (0.57–0.79)**	**1.01 (1.01–1.02)**
Adolescents 10–14 years	1.00 (0.99–1.01)	**0.64 (0.56–0.73)**	**1.01 (1.00–1.02)**
Adolescents 15–17 years	1.00 (0.99–1.01)	**0.61 (0.52–0.71)**	**1.01 (1.01–1.02)**
**Japan**	Broad-spectrum antibiotic prescription rate per 1000 children		
Overall	0.99 (0.98–0.99)	**0.46 (0.39–0.54)**	**1.03 (1.02–1.03)**
Male children	0.99 (0.98–0.99)	**0.47 (0.40–0.55)**	**1.03 (1.02–1.03)**
Female children	0.99 (0.98–0.99)	**0.45 (0.38–0.53)**	**1.03 (1.02–1.03)**
Children 0–1 years	0.99 (0.98–0.99)	**0.54 (0.43–0.67)**	**1.02 (1.01–1.03)**
Children 2–5 years	0.99 (0.98–0.99)	**0.45 (0.38–0.54)**	**1.02 (1.02–1.03)**
Children 6–9 years	0.99 (0.98–1.00)	**0.41 (0.33–0.51)**	**1.03 (1.02–1.04)**
Adolescents 10–14 years	0.99 (0.98–1.00)	**0.45** (**0.37–0.55)**	**1.03 (1.02–1.04)**
Adolescents 15–17 years	0.99 (0.98–1.00)	**0.51 (0.43–0.61)**	**1.02 (1.01–1.03)**
**Norway**	Proportion of prescriptions with broad-spectrum antibiotics		
Overall	**1.00 (1.00–1.01)**	**1.20 (1.11–1.30)**	1.00 (0.99–1.00)
Male children	**1.01 (1.00–1.01)**	**1.16 (1.08–1.24)**	0.99 (0.99–1.00)
Female children	**1.00 (1.00–1.01)**	**1.26 (1.14–1.39)**	1.00 (0.99–1.00)
Children 0–1 years	**1.02 (1.01–1.02)**	**1.51 (1.28–1.78)**	0.98 (0.98–0.99)
Children 2–5 years	**1.01 (1.00–1.01)**	**1.45 (1.23–1.72)**	0.99 (0.98–1.00)
Children 6–9 years	**1.01 (1.00–1.01)**	**1.37 (1.23–1.52)**	0.99 (0.99–1.00)
Adolescents 10–14 years	**1.00 (1.00–1.00)**	1.05 (0.95–1.16)	1.00 (0.99–1.00)
Adolescents 15–17 years	1.00 (0.99–1.00)	0.85 (0.78–0.92)	**1.01 (1.00–1.01)**
**Japan**	Proportion of prescriptions with broad-spectrum antibiotics		
Overall	1.00 (1.00–1.00)	**0.97 (0.95–0.98)**	1.00 (1.00–1.00)
Male children	1.00 (1.00–1.00)	**0.97 (0.95–0.98)**	1.00 (1.00–1.00)
Female children	1.00 (1.00–1.00)	**0.97 (0.96–0.99)**	1.00 (1.00–1.00)
Children 0–1 years	1.00 (1.00–1.00)	**0.97 (0.95–0.99)**	1.00 (1.00–1.00)
Children 2–5 years	1.00 (1.00–1.00)	**1.02 (1.00–1.03)**	1.00 (1.00–1.00)
Children 6–9 years	1.00 (1.00–1.00)	1.00 (0.99–1.02)	1.00 (1.00–1.00)
Adolescents 10–14 years	1.00 (1.00–1.00)	**0.93 (0.91–0.96)**	1.00 (1.00–1.00)
Adolescents 15–17 years	1.00 (1.00–1.00)	**0.86 (0.82–0.89)**	**1.01 (1.00–1.01)**
**Norway**	Proportion of antibiotic prescriptions with presumed bacterial diagnoses within the previous 7 days		
Overall	1.00 (1.00–1.00)	**0.90 (0.88–0.92)**	1.00 (1.00–1.01)
Male children	1.00 (1.00–1.00)	**0.95 (0.93–0.96)**	1.00 (1.00–1.00)
Female children	1.00 (1.00–1.00)	**0.83 (0.80–0.87)**	**1.01 (1.01–1.01)**
Children 0–1 years	1.00 (1.00–1.00)	**0.86 (0.82–0.91)**	1.01 (1.00–1.01)
Children 2–5 years	1.00 (1.00–1.00)	**0.90 (0.87–0.93)**	1.01 (1.00–1.01)
Children 6–9 years	1.00 (1.00–1.00)	**0.90 (0.87–0.94)**	1.01 (1.00–1.01)
Adolescents 10–14 years	1.00 (1.00–1.00)	**0.88 (0.85–0.91)**	1.00 (1.00–1.01)
Adolescents 15–17 years	1.00 (1.00–1.00)	**0.95 (0.92–0.98)**	1.00 (1.00–1.00)
**Japan**	Proportion of antibiotic prescriptions with presumed bacterial diagnoses within the previous 7 days		
Overall	1.00 (1.00–1.00)	**0.95 (0.93–0.97)**	1.00 (1.00–1.00)
Male children	1.00 (1.00–1.00)	**0.94 (0.92–0.96)**	1.00 (1.00–1.00)
Female children	1.00 (1.00–1.00)	**0.96 (0.94–0.98)**	1.00 (1.00–1.00)
Children 0–1 years	1.00 (1.00–1.00)	**0.93 (0.92–0.95)**	1.00 (1.00–1.00)
Children 2–5 years	1.00 (1.00–1.00)	**0.95 (0.93–0.97)**	1.00 (1.00–1.00)
Children 6–9 years	1.00 (1.00–1.00)	**0.95 (0.93–0.97)**	1.00 (1.00–1.00)
Adolescents 10–14 years	1.00 (1.00–1.00)	0.97 (0.95–1.00)	1.00 (1.00–1.00)
Adolescents 15–17 years	1.00 (1.00–1.00)	**0.95 (0.92–0.98)**	1.00 (1.00–1.00)

Bold font: statistically significant.

At the pandemic onset, both countries experienced a sharp decline in antibiotic use. Antibiotic prescription rates fell by 45% in Norway (RR: 0.55; 95% CI, 0.45–0.67) and 53% in Japan (RR: 0.47; 95% CI, 0.41–0.55). The most pronounced decreases were seen among children aged 2–5 in Norway and 6–9 in Japan (Figure 1 and Table 2). Following the initial drop, the rates gradually recovered, increasing by approximately 2% per month in both countries. Visually, by the end of 2023 the rates approached or slightly exceeded the predicted levels had the pandemic not occurred (Figure 1, Figure S1). Similar patterns were seen for broad-spectrum antibiotic prescription rates but to a lesser extent (Table 2; Figures S2 and S3).

#### Broad-spectrum antibiotic use

**Figure 2.**
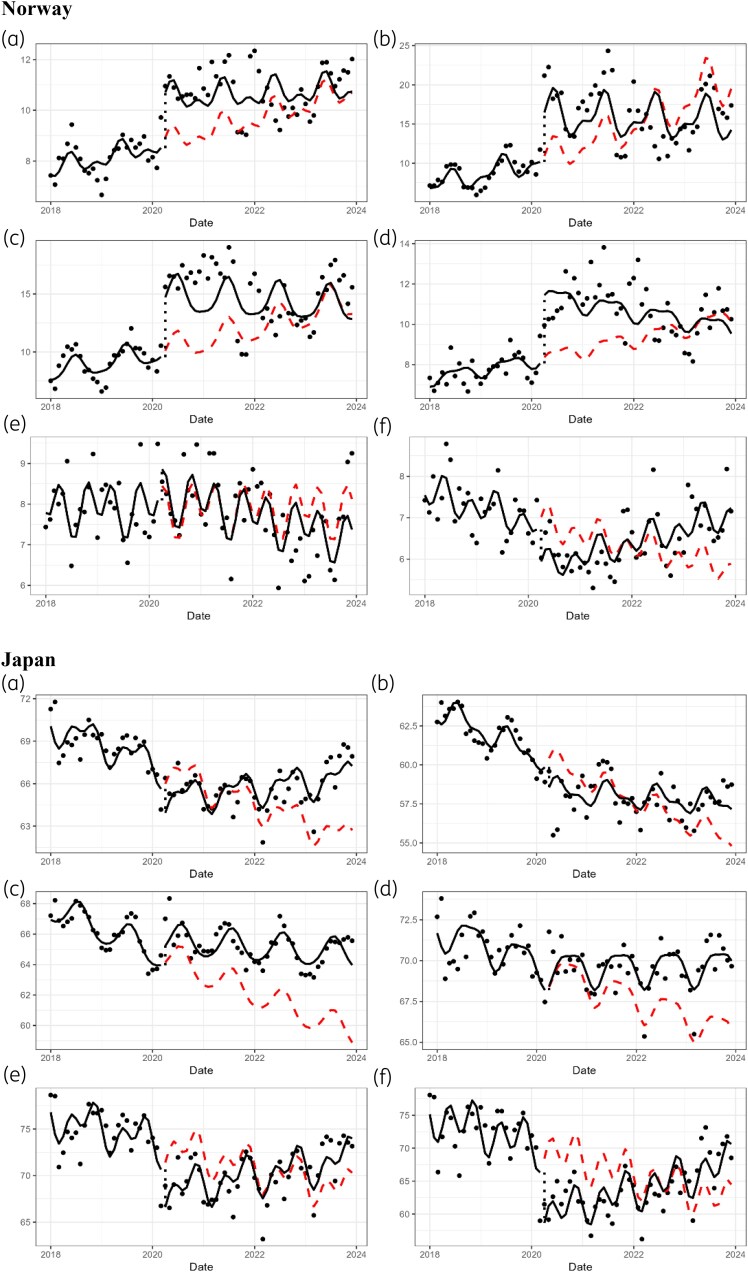
Proportion of antibiotic prescriptions with broad-spectrum antibiotics overall and by age groups in Norway (top) and Japan (bottom) (solid line: fitted values; dotted line: expected values had the pandemic not occurred). (a) Overall. (b) Children 0–1 years old. (c) Children 2–5 years old. (d) Children 6–9 years old. (e) Children 10–14 years old. (f) Adolescents 15–17 years old.

In Norway, broad-spectrum antibiotic prescriptions accounted for nearly 10% of all antibiotic prescriptions, with a slight increasing trend among younger age groups. A significant immediate increase of 20% in the proportion of broad-spectrum antibiotics was observed in March 2020 (RR: 1.20; 95% CI, 1.11–1.30), particularly among younger children. This elevated level persisted until the end of 2023 (Table 2; Figures 2 and  S4). Japan had a much higher baseline broad-spectrum use at around 65%–70%. A minor drop of 3% in the proportion of broad-spectrum antibiotics occurred at the onset of the pandemic (RR: 0.97; 95% CI, 0.95–0.98) followed by a slight increase until the end of 2023 (Table 2; Figures 2 and  S4).

#### Antibiotic prescriptions preceded by bacterial diagnoses

**Figure 3.**
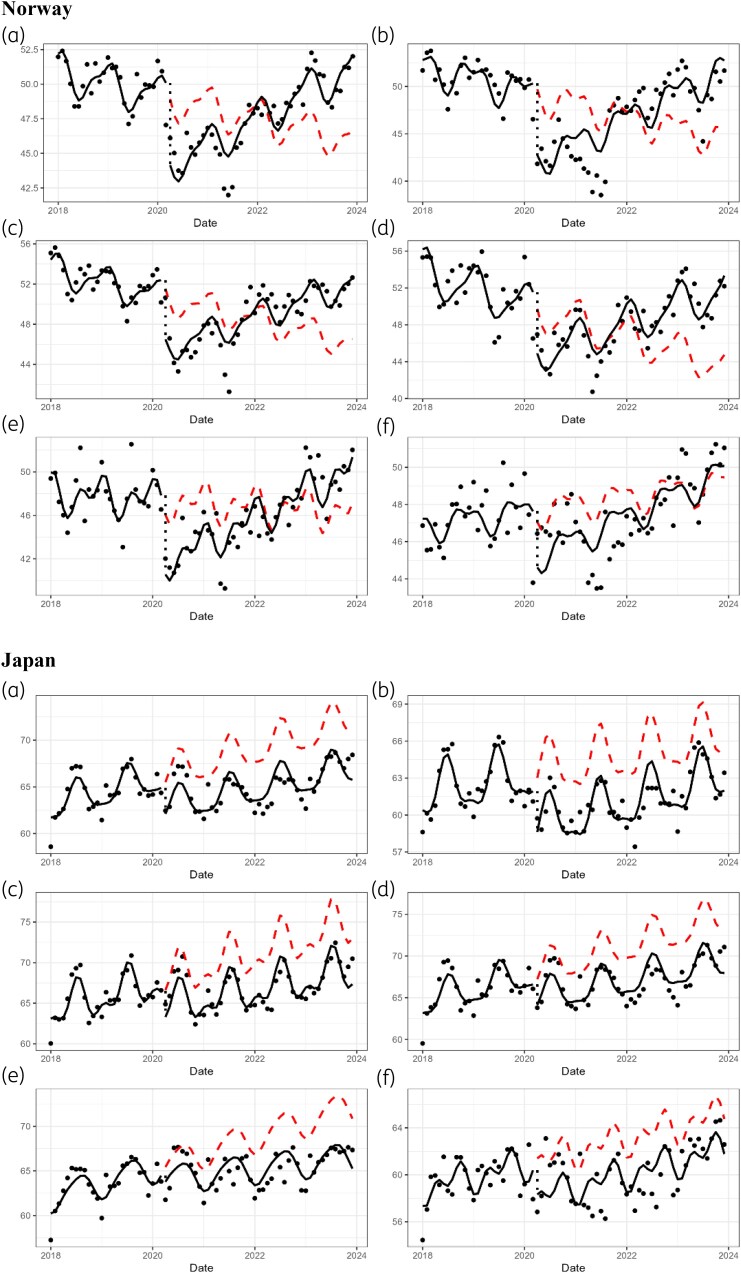
Proportion of antibiotic prescriptions with presumed bacterial infection diagnosis in the previous 7 days overall and by age groups in Norway (top) and Japan (bottom) (solid line: fitted values; dotted line: expected values had the pandemic not occurred). (a) Overall. (b) Children 0–1 years old. (c) Children 2–5 years old. (d) Children 6–9 years old. (e) Children 10–14 years old. (f) Adolescents 15–17 years old.

Japan had a slightly higher proportion of antibiotic prescriptions with bacterial diagnoses compared with Norway (65.0% versus 50.0%; Figures 3 and  S5). The proportions remained stable before March 2020 (Table 2). In March 2020, both countries experienced a modest drop (5% in Japan; 10% in Norway), followed by a gradual upward trend (Table 2; Figures 3 and  S5). By the end of 2023, the proportion in Japan remained below the predicted level had the pandemic not occurred, while Norway’s had slightly surpassed it (Figures 3 and  S5).

### Secondary outcomes

In Norway, outpatient visit rates (200–300/1000 children/month) were stable before the pandemic. An immediate increase of 28% occurred in March 2020 (RR: 1.28; 95% CI, 1.04–1.58), primarily among children over 6 years old, and this elevated rate persisted until the end of 2023 (Table S2; Figures S6 and S7). In contrast, Japan’s baseline visit rate (700–800/1000 children/month) was increasing but then dropped by 30% (RR: 0.70; 95% CI, 0.63–0.77) in March 2020, followed by a modest rebound (Table S2; Figures S6 and S7).

In Norway, visits with a bacterial diagnosis accounted for roughly 10% of all visits and were stable before March 2020. There were no major changes in the most common bacterial diagnoses across the years, and there were fewer respiratory infections recorded in 2020 in both countries (Table S4). In Japan, the rates accounted for nearly half of all visits and had been rising. Both countries experienced significant declines in visits with a bacterial diagnosis at the start of the pandemic: a 38% drop in Norway (RR: 0.62; 95% CI, 0.54–0.72) and 34% in Japan (RR: 0.64; 95% CI, 0.58–0.70), followed by gradual rebound until the end of 2023 (Table S2; Figures S8 and S9). The drop was more pronounced in male children and younger children in Norway (Table S2; Figures S8 and S9).

### Additional analyses

Extending the diagnostic look back period to 14 days did not substantially alter these findings (around70% in Japan; 50% in Norway; Table S3. When applying a relaxed definition of presumed bacterial infections, Norway's baseline level (60%-65%) become more comparable to that of Japan but similar pandemic related patterns were observed (Table S3).

## Discussion

### Antibiotic prescription and consultation rates

Historically, antibiotic prescription rates differed substantially between Norway and Japan, which suggests variations in healthcare-seeking behaviour, access to outpatient care or antibiotic prescribing norms. However, both countries experienced a sharp decrease in paediatric antibiotic use at the onset of the pandemic, which is consistent with prior evidence in other countries showing that outpatient antibiotic use declined broadly in 2020, attributed to reduced transmission of infectious diseases and potentially decreased healthcare seeking.^2,3^ Indeed, these patterns mirror those seen in the rates of visit with a diagnosed bacterial infection during the same period in both countries. Interestingly, overall consultation rates among children in Norway increased substantially between 2020 and 2023. This trend may have been driven by the rapid adoption of telehealth during the pandemic, as well as a heightened demand for medical care related to school reopenings and COVID-19 testing for travel.^17–19^ The pandemic’s unintended ‘natural experiment’ demonstrates how reduced transmission of common respiratory pathogens and/or restricted access to prescribers led to less antibiotic use among children. This finding suggests that stronger infection control initiatives could be effective in reducing antibiotic use. The rebound trend including resurgent rates exceeding pre-pandemic projections suggests the need for proactive reinforcement of prescribing guidelines, especially in those with high rates of antibiotic use (e.g. children aged 2–5 years). The rebound in overall antibiotic use was also found in several countries in Europe.^20^ Future studies should examine the drivers of this rebound, including changes in healthcare-seeking behaviour and clinical decision-making, and extended follow-up is needed. In addition, linking prescribing trends to downstream outcomes such as antimicrobial resistance rates would help quantify the clinical consequences of the observed changes.

### Broad-spectrum antibiotic use

In Norway, narrow-spectrum agents contributed to most of the antibiotic dispensations in accordance with national guidelines.^21^ In Japan, the top 90% of prescribed antibiotics comprises mostly broad-spectrum drugs, despite national guidelines resembling those of other countries.^22^ Variations in the level of medical training programmes and mandatory placements in infectious diseases for specialists might contribute to differential awareness of Norwegian and Japanese primary care physicians.^22^ Additionally, adverse reactions to penicillin (e.g. rash, diarrhoea) are relatively common in Japanese children, prompting clinicians to switch to cephalosporins in order to pre-empt parental complaints.^23^ The observed post-pandemic increase in broad-spectrum prescribing in Japan, alongside the significant rise in Norway, particularly among younger children, may reflect diagnostic uncertainty and a reliance on empirical treatment during the pandemic period. Furthermore, the pandemic likely discouraged people from seeking care for mild infections, reducing the number of patients with minor symptoms while increasing the proportion presenting with more severe illnesses who might require broad-spectrum agents. In Norway, with its low baseline of broad-spectrum antibiotic use, such changes would have been more apparent. In contrast, in Japan, where broad-spectrum prescribing was already common, the additional impact of the pandemic may have been less evident. Enhancing the adoption of diagnostic support tools, such as point-of-care testing, delayed prescription and evidence-based decision aids, may contribute to more judicious prescribing practices and a reduction in unnecessary broad-spectrum antibiotic use.^24^

### Antibiotic prescription fills/claims preceded by presumed bacterial infections

During the study period, about half (Norway) to two-thirds (Japan) of antibiotic prescription fills/claims were associated with prior recorded diagnosis of a presumed bacterial infection. These estimations are higher than prior estimates in other countries, especially in the USA (approximately 30%–40%), suggesting stronger cultures of diagnostic justification.^1,25^ Similarly to the USA, both countries experienced a modest decline in the proportion of visits with prior bacterial infections at the pandemic onset. However, the recovery seemed slower in the USA and Japan compared with Norway. The decline in antibiotic prescriptions associated with bacterial diagnoses likely captures both reduced clinical certainty and changes in diagnostic practices under pandemic pressure. Minimal differences were observed when applying 7 day and 14 day look-back periods in both countries, suggesting that the delayed or ‘wait-and-see’ approach (i.e. prescribers advise delaying antibiotic dispensing) was either infrequently used or typically implemented within a 7 day timeframe.

### Strengths and limitations

This study has several strengths. We utilized large-scale, high-quality, population-level data with a consistent analytical pipeline, which enhances the validity, reliability and reproducibility of the findings. Quasi-experimental designs with counterfactual estimation and an extended study period after the pandemic onset allowed us to measure and better understand the causal impact of the pandemic on key outcome measures. The cross-country design facilitated mutual learning between the two countries. We also employed a range of quantitative and qualitative outcome measures and did not rely solely on defined daily doses, which are not an appropriate metric of antibiotic use in paediatric populations.^26^ Some limitations of this study include the use of administrative data, which may not capture prescriptions that were issued but not dispensed and dispensed but not taken. Furthermore, we relied on the recorded diagnoses to determine whether an infection was viral or bacterial, thus misclassification bias might have occurred due to coding practice and the assignment of conditions to their presumed nature. In addition, it is known that the pandemic pressure led to many misdiagnoses and likely influenced prescribing behaviour.^27^ We also cannot account for changing parental behaviour, altered healthcare seeking, epidemiology, non-pharmaceutical interventions, prescribing behaviour or healthcare access dynamics during the study period, which may also have influenced the outcome measures.^27^ Notably, potential secular trends unrelated to COVID-19 might have influenced interrupted time series results (e.g. national antimicrobial campaigns) and could not be accounted for in our models. Towards the end of 2023, a few isolated outliers, likely corresponding to intense seasonal peaks of certain infectious diseases (e.g. mycoplasma pneumonia), were not fully captured by our models. As these outliers occurred well after the interruption point, they were unlikely to meaningfully influence the estimated pandemic impact. Additionally, the JMDC population might differ from the total Japanese population because of its insurer composition. However, we expect the resulting impact on our estimates for common acute paediatric infections to be limited because care-seeking for symptomatic children is unlikely to vary substantially across insurer schemes under Japan’s universal coverage. Lastly, we did not have access to the immigration and emigration data in Norway, thus the dispensations and visit rates were slightly overestimated. However, the annual net migration in Norway is less than 1% of the population, thus any resulting overestimation is expected to be minimal.

### Conclusions

The take-home messages of this study include: (i) the COVID-19 pandemic preceded an immediate and substantial reduction in paediatric antibiotic prescribing in both Norway and Japan, regardless of the level of use prior to the pandemic, followed by a gradual post-pandemic rebound; (ii) broad-spectrum antibiotic use was considerably higher in Japan than in Norway but was less affected by the pandemic; and (iii) sustained antibiotic stewardship efforts are needed to ensure appropriate paediatric antibiotic use in the post-pandemic era.

## Supplementary Material

dkag217_Supplementary_Data

## Data Availability

All data necessary to interpret the results and sufficient methodological detail to reproduce the analyses are provided in the manuscript. Due to Norwegian and Japanese data protection legislation, individual-level and aggregated data cannot be deposited in public repositories. However, data may be made available to researchers upon approval from the Regional Committees for Medical and Health Research Ethics (REC), compliance with the EU General Data Protection Regulation (GDPR), and approval from the respective data owners. Researchers seeking access to the data for replication purposes should apply through helsedata.no (Norway) and JMDC (Japan). All analyses were conducted using the publicly available R package *its2ae*, which is freely accessible to any researcher.

## Transparency declarations

T.F. was previously employed by the Department of Digital Health and Epidemiology at Kyoto University with support from Eisai Co., Ltd and Kyowa Kirin Co., Ltd; he has received research funds from AstraZeneca K.K. and the Pfizer Health Research Foundation, consulting fees from Asahi Kasei Pharma Corporation, JMDC Inc., MSD K.K., and Real World Data Co., Ltd, and honoraria from EPS Corporation, DeSC Healthcare, Inc., and the Research Institute of Healthcare Data Science; and his spouse is employed by MSD K.K. K.K. has received research funds from AstraZeneca K.K., Eisai Co., Ltd, Kyowa Kirin Co., Ltd, OMRON Corporation, and Toppan Inc; consulting fees from Advanced Medical Care Inc., JMDC Inc., LEBER Inc., Ubicom Holdings, Inc., Santen Pharmaceutical Co., Ltd and Shin Nippon Biomedical Laboratories Ltd; executive compensation from Cancer Intelligence Care Systems, Inc. and Mytreya Inc.; and honoraria from Kyoto University Original Co., Ltd, Pharma Business Academy Co., Ltd, Shionogi & Co., Ltd, Taisho Pharmaceutical Co., Ltd and SANSHO Co., Ltd. Other authors have nothing to disclose.

## Supplementary data

Figures S1 to S9, Tables S1 to S4 and Appendix S1 are available as Supplementary data at *JAC* Online.
