# Supplementary material for: Paediatric outpatient antibiotic utilization patterns and use of healthcare services before, during and after the COVID-19 pandemic: interrupted time series analysis using data from Norway and Japan
Source: J Antimicrob Chemother. 2026 Jun 18;81(7):dkag217. doi: 10.1093/jac/dkag217 (PMC13276868; doi:10.1093/jac/dkag217)
Supplement: dkag217_Supplementary_Data [file dkag217_supplementary_data.docx]

**Pediatric outpatient antibiotic utilization patterns and use of healthcare services before, during and after the COVID-19 pandemic: interrupted time series analysis using data from Norway and Japan**

Nhung TH Trinh, Toshiki Fukasawa, Takanori Yanai, Takayuki Okura, Atsushi Takayama, Takamasa Sakai, Olaug M Reiakvam, Hedvig ME Nordeng, Koji Kawakami

**APPENDICES**Table S1. Presumed bacterial conditions and associated ICD-10 and ICPC-2/ICPC-2B codes

Table S2. Impact of the pandemic on rate of pediatric visits and visits with a presumed bacterial infection diagnosis (rate ratio with 95% confidence interval) using March 2020 as the interruption point and pre-pandemic trend/level as reference

Table S3. Impact of the pandemic on the proportion of antibiotic prescriptions accompanied by a presumed bacterial infection diagnosis with different definitions (rate ratio with 95% confidence interval) using March 2020 as the interruption point and pre-pandemic trend/level as reference

Table S4. Top 10 presumed bacterial infections recorded by year

Figure S1. Antibiotic prescription rate in overall and by sex (black line: fitted values, red dotted line: predicted values had the pandemic not occurred): number of antibiotic prescription fills/claims per 1000 children per month

Figure S2. Broad-spectrum antibiotic prescription rate in overall and by age groups (black line: fitted values, red dotted line: predicted values had the pandemic not occurred): number of broad-spectrum antibiotic prescription fills/claims per 1000 children per month

Figure S3. Broad-spectrum antibiotic prescription rate in overall and by sex (black line: fitted values, red dotted line: predicted values had the pandemic not occurred): number of broad-spectrum antibiotic prescription fills/claims per 1000 children per month

Figure S4. Proportion of antibiotic prescriptions with broad-spectrum antibiotics in overall and by sex (black line: fitted values, red dotted line: predicted values had the pandemic not occurred)

Figure S5. Proportion of antibiotic prescriptions with presumed bacterial infection diagnosis in the previous 7 days in overall and by sex (black line: fitted values, red dotted line: predicted values had the pandemic not occurred)

Figure S6. Rate of pediatric visit per 1000 children per month in overall and by age groups (black line: fitted values, red dotted line: predicted values had the pandemic not occurred)

Figure S7. Rate of pediatric visit per 1000 children per month in overall and by sex (black line: fitted values, red dotted line: predicted values had the pandemic not occurred)

Figure S8. Rate of visits with a presumed bacterial infection diagnosis per 1000 children per month in overall and by age groups (black line: fitted values, red dotted line: predicted values had the pandemic not occurred)

Figure S9. Rate of visits with a presumed bacterial infection diagnosis per 1000 children per month in overall and by sex (black line: fitted values, red dotted line: predicted values had the pandemic not occurred)

**Appendix 1. Reporting checklist following STROBE guidelines for interrupted time series analysis**

| Section | Item no. | Description | Evaluation |
| --- | --- | --- | --- |
| Title and abstract | 1 | Indicate the study design (interrupted time series) in the title or abstract | yes |
| Background/rationale | 2 | Provide background regarding the intervention and setting under investigation to support the study rationale and methods | Yes |
| Objectives | 3 | 1. State specific objectives and any prespecified hypotheses 2. Distinguish between primary and secondary objectives | Yes |
| Intervention | 4 | Define the intervention time point(s) used in the analysis | Yes |
| Participants | 5 | 1. List eligibility criteria and methods of selection 2. Define subgroups 3. Consider including a comparison group not exposed to the intervention as a secondary group of participants | Yes  c is not relevant |
| Data sources and measurement | 6 | 1. List data source(s) 2. Comment on data completeness, validity, and changes in data coverage over time | Yes |
| Variables | 7 | 1. Define all variables 2. Outcome variable(s) 3. Descriptive and stratifying variable(s) 4. Comment on change in variable coding over time 5. Consider including details of variable coding in supplemental material, for example, appendix or research Web site | Yes  d and e are not applicable |
| Statistical methods | 8 | 1. Report all statistical methods   Study time intervals, for example, monthly, quarterly  Regression model, for example, ARIMA, linear, segmented  For ARIMA models, indicate the intervention function, for example, point, ramp, or step  Indicate the appropriateness of linear model(s) when applied  Number of preintervention, postintervention, and between intervention data points   1. Define the study period and number of preintervention data points used in forecasting 2. Indicate how autocorrelation, nonstationarity, and seasonality were tested and handled 3. Consider a lag period if intervention effects are gradual or delayed 4. Define and distinguish between primary and secondary or sensitivity analyses 5. Consider use of comparison outcome(s) and/or population(s) not exposed to the intervention(s) as secondary analyses 6. Report statistical software used for analysis | Yes  d and f are not applicable |
| Participants | 9 | 1. Report the number of individuals and/or observations in each group analyzed 2. Consider use of a flow diagram 3. Describe characteristics and indicate missing data | Yes  b is not applicable |
| Outcome data | 10 | 1. Report the number of outcomes examined over the study period 2. Report the average, minimum, and maximum number of outcomes across time intervals 3. Report on data variability 4. Comment on outliers and ceiling or floor effects where relevant | Yes  d is not applicable |
| Main results | 11 | 1. Present results using a graphical display with intervention time point(s) clearly defined 2. Consider including forecasted results graphically 3. Report absolute and/or relative change(s) and their significance, for example, clinical or policy and statistical | Yes |
| Other analyses | 12 | Report additional results (secondary and sensitivity analyses) in the article, appendix, or research Web site | Yes |
| Key results | 13 | Summarize key results with reference to study objectives | Non applicable |
| Context | 14 | 1. Provide context related to possible confounding 2. Discuss relevant cointerventions that occurred during the study period 3. Comment on the stability of participant characteristics over time 4. Comment on the stability of outcome coding over time 5. Discuss results of comparison analyses or provide a rationale if no comparison group was considered | Yes  d and e are not applicable |
| Limitations | 15 | 1. Discuss limitations of the study 2. Comment on data variability and appropriateness of the number of data points 3. Comment on ceiling or floor effects and outliers where relevant 4. Discuss direction and magnitude of any potential bias | Yes |
| Interpretation | 16 | Provide overall interpretation of results considering objectives, limitations, results from similar studies, and other relevant evidence | Yes |
| Funding | 17 | List funding source(s) and role of funders | Yes |
| References | 18 | Reference methodological articles that support statistical methods used | Yes |

**Table S1. Presumed bacterial conditions and associated ICD-10 and ICPC-2/ICPC-2B codes**

| **Categories** | **Sub-group** | **ICD 10** | **ICPC-2/ICPC-2B** |
| --- | --- | --- | --- |
| Potentially bacterial RTIs | Pneumonia | A37 Whooping cough  B96.0 Mycoplasma pneumoniae [M. pneumoniae]  J13 Pneumonia due to Streptococcus pneumoniae  J14 Pneumonia due to Haemophilus influenzae  J15 Bacterial pneumonia, not elsewhere classified  J16 Pneumonia due to other infectious organisms, not elsewhere classified  J17 Pneumonia in diseases classified elsewhere  J18 Pneumonia, organism unspecified | R71 Whooping cough  R81 Pneumonia  *Additional ICPC-2B codes:*  A78.0198 |
|  | Pharyngitis | **Acute**  A38 Scarlet fever  A69 Other Vincent infections  J02 Acute pharyngitis  J03 Acute tonsillitis  J36 Peritonsillar abscess  R07.0 Sore throat  **Chronic**  J35.0 Chronic tonsilitis and adenoiditis | R72 Strep throat  R76 Tonsillitis acute  R90 Hypertrophy tonsils/adenoids  R21 Throat symptom/complaint  *Additional ICPC-2B codes:*  A78.0008  A78.0017  A78.0018  A78.0020  A78.0039  A78.0060  A78.0066  A78.0117  D83.0003  D83.0008  D83.0035  D83.0037  R90.0004 |
|  | Sinusitis | **Acute**  J01 Acute sinusitis  J34.8 Other specified disorders of nose and nasal sinuses  **Chronic**  J32 Chronic sinusitis | R75 Sinusitis acute/chronic  *Additional ICPC-2B codes:*  R99.0016  R99.0017 |
|  | Otitis | **Acute**  H65 Nonsuppurative otitis media  H66 Suppurative and unspecified otitis media  H70 Mastoiditis and related conditions  H92 Otalgia and effusion of ear  H73.0 Acute myringitis  **Chronic**  H73.1 Chronic myringitis | H71 Acute otitis media/myringitis  H72 Serous otitis media  H74 Chronic otitis media  *Additional ICPC-2B codes:*  H01.0000- H01.0002  H04.0000- H04.0003  H05.0000- H05.0002 |
| Presumed bacterial non-RTIs | Urinary tract  infections | N12 Tubulo-interstitial nephritis, not specified as acute or chronic idem  N15.1 Renal and perinephric abscess  N30 Cystitis  N34 Urethritis  N39.0 Urinary tract infection, site not specified  N70.0 Acute salpingitis and oophoritis  N72 Inflammatory disease of cervix uteri  N73 Pelvic peritonitis and pelvic cellulitis  N75.0 Abscess of Bartholin's gland | U70 Pyelonephritis/pyelitis  U71 Cystitis/urinary infection other  U72 Urethritis  X74 Pelvic inflammatory disease  *Additional ICPC-2B codes:*  X85.0001  X85.0002  X99.0005 |
|  | Gastrointestinal  Infections | A00-A09 except A08 Intestinal infectious diseases except viral and other specified intestinal infections  B98.0 - Helicobacter pylori [H. pylori] | D70 Gastrointestinal infection  *Additional ICPC-2B codes:*  A78.0161 |
|  | Skin and soft tissue infections | H00 – Hordeolum and chalazion  H60 Otitis externa  I88 Nonspecific lymphadenitis  I89.1 Lymphangitis  J34.0 Abscess, furuncle and carbuncle of nose  K04.7 Periapical abscess without sinus  L01 Impetigo  L02 Cutaneous abscess, furuncle and carbuncle  L03 Cellulitis  L04 Acute lymphadenitis  L08 Other local infections of skin and subcutaneous tissue  L30 Other dermatitis  L66.2 Folliculitis decalvans  L70 Acne  M00-M03 Infectious arthropathies  M46.2 Osteomyelitis of vertebra  M46.3 Infection of intervertebral disc (pyogenic)  M46.4 Discitis, unspecified  M46.5 Other infective spondylopathies  M86 Osteomyelitis  M72.6 Necrotizing fasciitis  P38 - Omphalitis of newborn  P39.0 Neonatal infective mastitis | F72 Blepharitis/stye/chalazion  H70 Otitis externa  B71 Lymphadenitis non-specific  R73 Boil/abscess nose  S84 Impetigo  S10 Boil/carbuncle  S09 Infected finger/toe  S76 Skin infection other  B70 Lymphadenitis acute  S98 Urticaria  S96 Acne  L70 Infections musculoskeletal system  L87 Bursitis/tendinitis/synovitis NOS  *Additional ICPC-2B codes:*  B99.0010  D82.0058  S88.0000  S88.0002  S88.0003  S88.0005  S88.0007  S88.0018  S88.0028  S92.0001  S92.0004  S99.0034  S99.0054  S23.0016  A94.0023  L99.0134  L99.0166  L87.0175  A94.0225  L99.0007- L99.0012  L99.0326- L99.0335  L99.0473- L99.0483  L99.0526- L99.0535  L99.0928- L99.0957  L99.1288- L99.1298  L99.1322- L99.1342  L99.1652- L99.1661 |
|  | Miscellaneous  bacterial  infections | A15-A19 Tuberculosis  A20-A28 Certain zoonotic bacterial diseases  A30-A49 Other bacterial disease  A50-A58 Bacterial sexually transmitted infections  A65–A69: Other Spirochetal Diseases  A70-A74 Other diseases caused by chlamydiae  A75-A79 Rickettsioses  B95 Streptococcus, Staphylococcus, and Enterococcus as the cause of diseases classified elsewhere  B90 Sequelae of tuberculosis  B96 Other bacterial agents as the cause of diseases classified elsewhere  G00-G07 Meningitis, encephalitis, intraspinal abscess  H68- Eustachian salpingitis and obstruction  K04 - Diseases of pulp and periapical tissues  K05 - Gingivitis and periodontal diseases  K08 - Other disorders of teeth and supporting structures  K11.2 – Sialoadenitis  K35 - Acute appendicitis  K37 - Unspecified appendicitis  T85.7 - Infection and inflammatory reaction due to other internal prosthetic devices, implants and grafts | Y70 Syphilis male  X70 Syphilis female  Y71 Gonorrhoea male  X71 Gonorrhoea female  S76 Skin infection other  F86 Trachoma  F70 Conjunctivitis infectious  A70 Tuberculosis  N71 Meningitis/encephalitis  H73 Eustachian salpingitis  D88 Appendicitis  *Additional ICPC-2B codes:*  R83.0003  R83.0016  R83.0018  R83.0022  R83.0023  R83.0027  N72.0000- N72.0003  Y99.0010  Y99.0011  X99.0043  X99.0054  Y99.0034  U72.0004  X92.0000- X92.0007  Y74.0005  Y74.0006  X99.0006  X99.0070  Y99.0002  Y99.0033  Y99.0007  D83.0003  D83.0008  D83.0035  D83.0037  R81.0008  A78.0001- A78.0006  A78.0008  A78.0009  A78.0016- A78.0022  A78.0026  A78.0030  A78.0034  A78.0036  A78.0039-A78.0043  A78.0045- A78.0048  A78.0053  A78.0056- A78.0062  A78.0066  A78.0068-A78.0071  A78.0079  A78.0082  A78.0084  A78.0085  A78.0090- A78.0099  A78.0106- A78.0108  A78.0111- A78.0113  A78.0115- A78.0120  A78.0124- A78.0130  A78.0132- A78.0144  A78.0147-A78.0153  A78.0155  A78.0160  A78.0164- A78.0168  A78.0173  A78.0178  A78.0179  A78.0185  A78.0186- A78.0188  A78.0192  A78.0193  A78.0195  A78.0196  A78.0198  A78.0199  A78.0200  A78.0205  A78.0207  A78.0208  A78.0209  A78.0212  A78.0234  A78.0237- A78.0258  A78.0260  A78.0263  A78.0264  A78.0265  A78.0270- A78.0272  A78.0280- A78.0282  N73.0001  N73.0011-N73.0013  H73.0000  H73.0002- H73.0004  D83.0024  A89.0026  D82.0074  D19.0000  D19.0006  D82.0000  D82.0001  D82.0003  D82.0005  D82.0006  D82.0010- D82.0012  D82.0016- D82.0018  D82.0020  D82.0023  D82.0031  D82.0039  D82.0047  D82.0050  D82.0053  D82.0057  D82.0058  D82.0060-D82.0062  D82.0073  D82.0076 |

**Table S2. Impact of the pandemic on rate of pediatric visits and visits with a presumed bacterial infection diagnosis (rate ratio with 95% confidence interval)** using March 2020 as the interruption point and pre-pandemic trend/level as reference

|  | **Pre-pandemic trend** | **Change in level** | **Change in slope** |
| --- | --- | --- | --- |
| **Norway** | Rate of pediatric visits | | |
| Overall | 1.00 (0.99-1.00) | **1.28 (1.04-1.58)** | 1.00 (0.99-1.01) |
| Male children | 1.00 (0.99-1.00) | **1.29 (1.04-1.58)** | 1.00 (0.99-1.01) |
| Female children | 1.00 (0.99-1.00) | **1.27 (1.03-1.57)** | 1.00 (0.99-1.01) |
| Children 0-1 years | 1.00 (1.00-1.00) | 0.96 (0.79-1.15) | **1.01 (1.00-1.01)** |
| Children 2-5 years | 1.00 (1.00-1.00) | 1.08 (0.90-1.31) | 1.00 (1.00-1.01) |
| Children 6-9 years | 1.00 (0.99-1.00) | **1.36 (1.08-1.70)** | 1.00 (0.98-1.01) |
| Adolescents 10-14 years | 1.00 (0.98-1.01) | **1.54 (1.17-2.03)** | 1.00 (0.98-1.01) |
| Adolescents 15-17 years | 1.00 (0.99-1.00) | **1.34 (1.06-1.70)** | 1.00 (0.99-1.01) |
| **Japan** | Rate of pediatric visits | | |
| Overall | 1.00 (0.99-1.00) | **0.70 (0.63-0.77)** | **1.01 (1.01-1.02)** |
| Male children | 1.00 (0.99-1.00) | **0.70 (0.64-0.77)** | **1.01 (1.01-1.02)** |
| Female children | 1.00 (0.99-1.00) | **0.69 (0.63-0.77)** | **1.01 (1.01-1.02)** |
| Children 0-1 years | 1.00 (1.00-1.00) | **0.70 (0.63-0.78)** | **1.01 (1.01-1.02)** |
| Children 2-5 years | 1.00 (0.99-1.00) | **0.63 (0.57-0.71)** | **1.02 (1.01-1.02)** |
| Children 6-9 years | 1.00 (0.99-1.00) | **0.67 (0.59-0.75)** | **1.02 (1.01-1.02)** |
| Adolescents 10-14 years | 1.00 (0.99-1.00) | **0.77 (0.68-0.86)** | **1.01 (1.01-1.02)** |
| Adolescents 15-17 years | 1.00 (0.99-1.00) | **0.82 (0.73-0.92)** | **1.01 (1.01-1.02)** |
| **Norway** | Rate of visits with presumed bacterial infection diagnosis | | |
| Overall | 1.00 (0.99-1.00) | **0.62 (0.54-0.72)** | **1.02 (1.01-1.03)** |
| Male children | 1.00 (0.99-1.00) | **0.66 (0.58-0.75)** | **1.02 (1.01-1.02)** |
| Female children | 1.00 (0.99-1.00) | **0.58 (0.5-0.67)** | **1.02 (1.01-1.03)** |
| Children 0-1 years | 1.00 (0.99-1.00) | **0.48 (0.36-0.63)** | **1.03 (1.02-1.04)** |
| Children 2-5 years | 1.00 (0.99-1.00) | **0.48 (0.36-0.63)** | **1.03 (1.02-1.04)** |
| Children 6-9 years | 1.00 (0.99-1.00) | **0.59 (0.50-0.69)** | **1.02 (1.01-1.03)** |
| Adolescents 10-14 years | 1.00 (0.99-1.00) | **0.59 (0.50-0.69)** | **1.02 (1.01-1.03)** |
| Adolescents 15-17 years | 1.00 (1.00-1.01) | 0.81 (0.61-1.07) | **1.01 (1.00-1.02)** |
| **Japan** | Rate of visits with presumed bacterial infection diagnosis | | |
| Overall | 1.00 (0.99-1.00) | **0.64 (0.58-0.70)** | **1.01 (1.01-1.02)** |
| Male children | 1.00 (0.99-1.00) | **0.64 (0.58-0.70)** | **1.01 (1.01-1.02)** |
| Female children | 1.00 (0.99-1.00) | **0.64 (0.58-0.71)** | **1.01 (1.01-1.02)** |
| Children 0-1 years | 1.00 (1.00-1.00) | **0.69 (0.62-0.77)** | **1.01 (1.01-1.02)** |
| Children 2-5 years | 1.00 (0.99-1.00) | **0.58 (0.52-0.65)** | **1.02 (1.01-1.02)** |
| Children 6-9 years | 1.00 (0.99-1.00) | **0.58 (0.51-0.67)** | **1.02 (1.01-1.02)** |
| Adolescents 10-14 years | 1.00 (0.99-1.00) | **0.58 (0.51-0.67)** | **1.02 (1.01-1.02)** |
| Adolescents 15-17 years | 1.00 (1.00-1.01) | **0.80 (0.71-0.89)** | **1.01 (1.00-1.01)** |

Bold font: statistically significant

**Table S3. Impact of the pandemic on the proportion of antibiotic prescriptions accompanied by a presumed bacterial infection diagnosis with different definitions (rate ratio with 95% confidence interval)** using March 2020 as the interruption point and pre-pandemic trend/level as reference

|  | **Pre-pandemic trend** | **Change in level** | **Change in slope** |
| --- | --- | --- | --- |
| **Norway** | With presumed bacterial diagnoses (14 days) | | |
| Overall | 1.00 (1.00-1.00) | **0.91 (0.89-0.93)** | 1.00 (1.00-1.01) |
| Children 0-1 years | 1.00 (1.00-1.00) | **0.86 (0.82-0.91)** | **1.01 (1.00-1.01)** |
| Children 2-5 years | 1.00 (1.00-1.00) | **0.90 (0.88-0.93)** | **1.01 (1.00-1.01)** |
| Children 6-9 years | 1.00 (1.00-1.00) | **0.91 (0.87-0.94)** | **1.01 (1.00-1.01)** |
| Adolescents 10-14 years | 1.00 (1.00-1.00) | **0.89 (0.86-0.92)** | 1.00 (1.00-1.01) |
| Adolescents 15-17 years | 1.00 (1.00-1.00) | **0.96 (0.92-0.99)** | 1.00 (1.00-1.00) |
| **Japan** | With presumed bacterial diagnoses (14 days) | | |
| Overall | 1.00 (1.00-1.00) | **0.95 (0.93-0.97)** | 1.00 (1.00-1.00) |
| Children 0-1 years | 1.00 (1.00-1.00) | **0.93 (0.92-0.95)** | 1.00 (1.00-1.00) |
| Children 2-5 years | 1.00 (1.00-1.00) | **0.95 (0.93-0.97)** | 1.00 (1.00-1.00) |
| Children 6-9 years | 1.00 (1.00-1.00) | **0.95 (0.93-0.97)** | 1.00 (1.00-1.00) |
| Adolescents 10-14 years | 1.00 (1.00-1.00) | 0.97 (0.95-1.00) | 1.00 (1.00-1.00) |
| Adolescents 15-17 years | 1.00 (1.00-1.00) | **0.95 (0.92-0.98)** | 1.00 (1.00-1.00) |
| **Norway** | with bacterial diagnoses (relaxed definition – 7 days) | | |
| Overall | 1.00 (1.00-1.00) | **0.83 (0.79-0.87)** | **1.01 (1.00-1.01)** |
| Children 0-1 years | 1.00 (1.00-1.00) | **0.84 (0.79-0.88)** | **1.01 (1.00-1.01)** |
| Children 2-5 years | 1.00 (1.00-1.00) | **0.84 (0.80-0.89)** | **1.01 (1.00-1.01)** |
| Children 6-9 years | 1.00 (1.00-1.00) | **0.82 (0.78-0.87)** | **1.01 (1.01-1.01)** |
| Adolescents 10-14 years | 1.00 (1.00-1.00) | **0.89 (0.86-0.93)** | 1.00 (1.00-1.01) |
| Adolescents 15-17 years | 1.00 (1.00-1.00) | **0.86 (0.83-0.89)** | 1.00 (1.00-1.01) |

Bold font: statistically significant

**Table S4. Top 10 presumed bacterial infections recorded by year (number of consultations)**

| **2018** | **2019** | **2020** | **2021** | **2022** | **2023** |
| --- | --- | --- | --- | --- | --- |
| **Norway** | | | | | |
| \| H71 \| ICPC-2 \| 60726 \| \| --- \| --- \| --- \| \| F70 \| ICPC-2 \| 48760 \| \| U71 \| ICPC-2 \| 33594 \| \| R21 \| ICPC-2 \| 28411 \| \| S96 \| ICPC-2 \| 28191 \| \| R76 \| ICPC-2 \| 18691 \| \| R72 \| ICPC-2 \| 18549 \| \| H65.3 \| ICD-10 \| 16945 \| \| H65.2 \| ICD-10 \| 15650 \| \| S84 \| ICPC-2 \| 14932 \| | \| H71 \| ICPC-2 \| 58609 \| \| --- \| --- \| --- \| \| F70 \| ICPC-2 \| 46936 \| \| U71 \| ICPC-2 \| 32919 \| \| S96 \| ICPC-2 \| 30540 \| \| R21 \| ICPC-2 \| 29012 \| \| R72 \| ICPC-2 \| 19974 \| \| R76 \| ICPC-2 \| 17853 \| \| H65.3 \| ICD-10 \| 15618 \| \| H65.2 \| ICD-10 \| 14701 \| \| S84 \| ICPC-2 \| 14601 \| | \| S96 \| ICPC-2 \| 33145 \| \| --- \| --- \| --- \| \| U71 \| ICPC-2 \| 31042 \| \| H71 \| ICPC-2 \| 30873 \| \| R21 \| ICPC-2 \| 25862 \| \| F70 \| ICPC-2 \| 21505 \| \| S98 \| ICPC-2 \| 12472 \| \| R72 \| ICPC-2 \| 12204 \| \| S84 \| ICPC-2 \| 11507 \| \| H65.2 \| ICD-10 \| 9931 \| \| R76 \| ICPC-2 \| 9788 \| | \| S96 \| ICPC-2 \| 33635 \| \| --- \| --- \| --- \| \| H71 \| ICPC-2 \| 33565 \| \| U71 \| ICPC-2 \| 29566 \| \| F70 \| ICPC-2 \| 24176 \| \| R21 \| ICPC-2 \| 23633 \| \| S98 \| ICPC-2 \| 12213 \| \| L70.0 \| ICD-10 \| 10026 \| \| S84 \| ICPC-2 \| 9940 \| \| R72 \| ICPC-2 \| 9092 \| \| F72 \| ICPC-2 \| 8717 \| | \| F70 \| ICPC-2 \| 57346 \| \| --- \| --- \| --- \| \| H71 \| ICPC-2 \| 52368 \| \| S96 \| ICPC-2 \| 33381 \| \| R21 \| ICPC-2 \| 31207 \| \| U71 \| ICPC-2 \| 28100 \| \| R72 \| ICPC-2 \| 18821 \| \| S98 \| ICPC-2 \| 13752 \| \| R76 \| ICPC-2 \| 13111 \| \| H65.3 \| ICD-10 \| 11938 \| \| L70.0 \| ICD-10 \| 11129 \| | \| H71 \| ICPC-2 \| 61128 \| \| --- \| --- \| --- \| \| F70 \| ICPC-2 \| 42834 \| \| S96 \| ICPC-2 \| 40357 \| \| R21 \| ICPC-2 \| 32312 \| \| R72 \| ICPC-2 \| 30247 \| \| U71 \| ICPC-2 \| 27109 \| \| H65.3 \| ICD-10 \| 15291 \| \| S84 \| ICPC-2 \| 14532 \| \| L70.0 \| ICD-10 \| 14169 \| \| R76 \| ICPC-2 \| 13476 \| |
| **Japan** | | | | | |
| \| L30.9 \| 1443893 \| \| --- \| --- \| \| J02.9 \| 1017281 \| \| A09.9 \| 942858 \| \| J01.9 \| 921743 \| \| J32.9 \| 621448 \| \| H66.9 \| 350462 \| \| H65.9 \| 335425 \| \| H60.5 \| 287739 \| \| L70.0 \| 261549 \| \| J03.9 \| 213983 \| | \| L30.9 \| 1697362 \| \| --- \| --- \| \| J02.9 \| 1132414 \| \| A09.9 \| 1017598 \| \| J01.9 \| 1005953 \| \| J32.9 \| 690922 \| \| H66.9 \| 377026 \| \| H65.9 \| 368388 \| \| L70.0 \| 328718 \| \| H60.5 \| 319255 \| \| J03.9 \| 240183 \| | \| L30.9 \| 1696546 \| \| --- \| --- \| \| J02.9 \| 672939 \| \| J01.9 \| 668305 \| \| A09.9 \| 588458 \| \| J32.9 \| 522278 \| \| L70.0 \| 456294 \| \| H60.5 \| 273453 \| \| H65.9 \| 263683 \| \| L08.9 \| 233975 \| \| H66.9 \| 212357 \| | \| L30.9 \| 1949019 \| \| --- \| --- \| \| J02.9 \| 792140 \| \| J01.9 \| 741749 \| \| A09.9 \| 711152 \| \| J32.9 \| 563686 \| \| L70.0 \| 524981 \| \| H60.5 \| 327239 \| \| H65.9 \| 265634 \| \| L08.9 \| 257301 \| \| H66.9 \| 250631 \| | \| L30.9 \| 2061186 \| \| --- \| --- \| \| J02.9 \| 1063213 \| \| A09.9 \| 942258 \| \| J01.9 \| 758732 \| \| J32.9 \| 554366 \| \| L70.0 \| 536480 \| \| H60.5 \| 353010 \| \| L08.9 \| 274923 \| \| H66.9 \| 251431 \| \| H65.9 \| 242521 \| | \| L30.9 \| 2199008 \| \| --- \| --- \| \| J02.9 \| 1577878 \| \| A09.9 \| 1281300 \| \| J01.9 \| 1114115 \| \| J32.9 \| 705609 \| \| L70.0 \| 558089 \| \| H66.9 \| 422176 \| \| H60.5 \| 421606 \| \| H65.9 \| 324455 \| \| J03.9 \| 308961 \| |

Note: H71 (ICPC-2, Otitis externa), F70 (ICPC-2, conjunctivitis infectious), U71 (ICPC-2, Cystitis), R21 (ICPC-2, throat symptoms), S96 (ICPC-2, acne), R76 (ICPC-2, Tonsilitis acute), R72 (ICPC-2, Strep throat), H65.3 (ICD-10, Chronic mucoid otitis media), H65.2 (ICD-10, Chronic serous otitis media), S84 (ICPC-2, Impetigo), S98 (ICPC-2, Urticaria), L70.0 (ICD-10, acne vulgaris), F72 (ICPC-2, Blepharitis), L30.9 (ICD-10, Dermatitis), J02.9 (ICD-10, Acute pharyngitis), A09.9 (ICD-10, infectious gastroenteritis and colitis), J01.9 (ICD-10, acute sinusitis), J32.9 (chronic sinusitis), H66.9 (ICD-10, otitis media), H65.9 (ICD-10, nonsuppurative otitis media), H60.5 (ICD-10, acute noninfective otitis externa), J03.9 (ICD-10, acute tonsilitis), L08.9 (ICD-10, local skin infection)

**Figure S1. Antibiotic prescription rate in overall and by sex (black line: fitted values, red dotted line: predicted values had the pandemic not occurred): number of antibiotic prescription fills/claims per 1000 children per month**

| **Norway**  **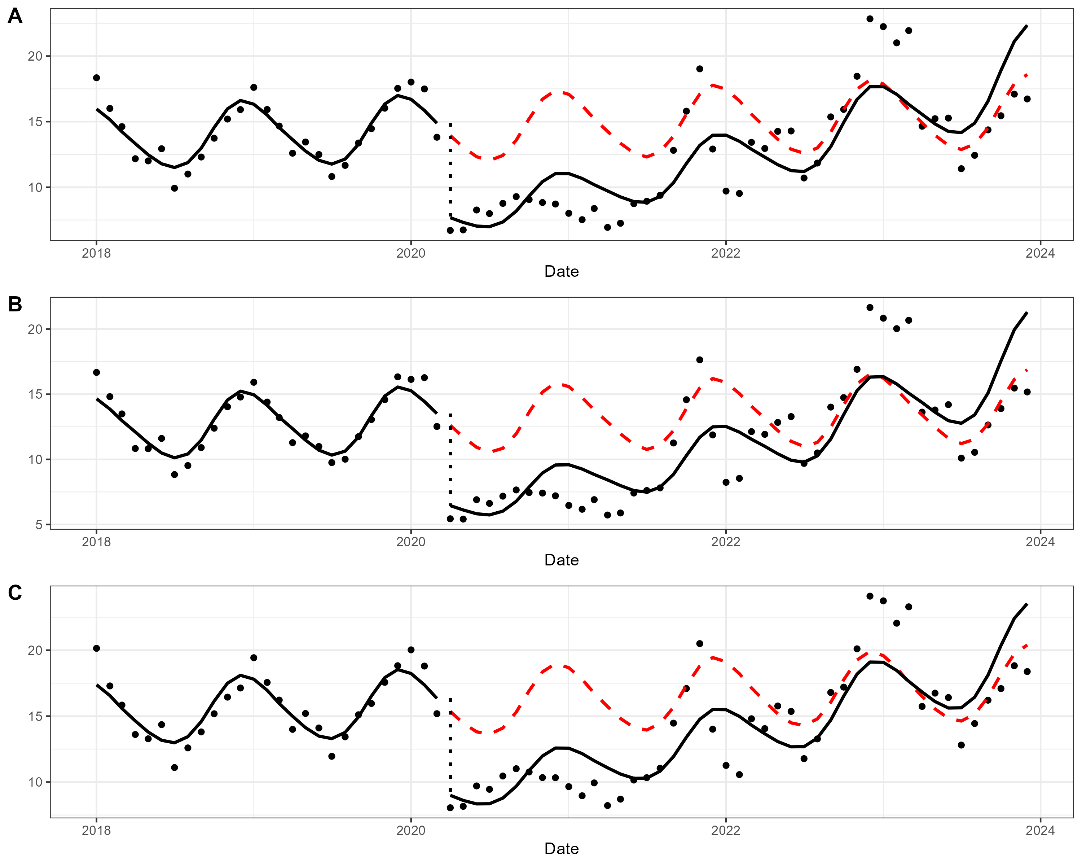** |
| --- |
| **Japan**  **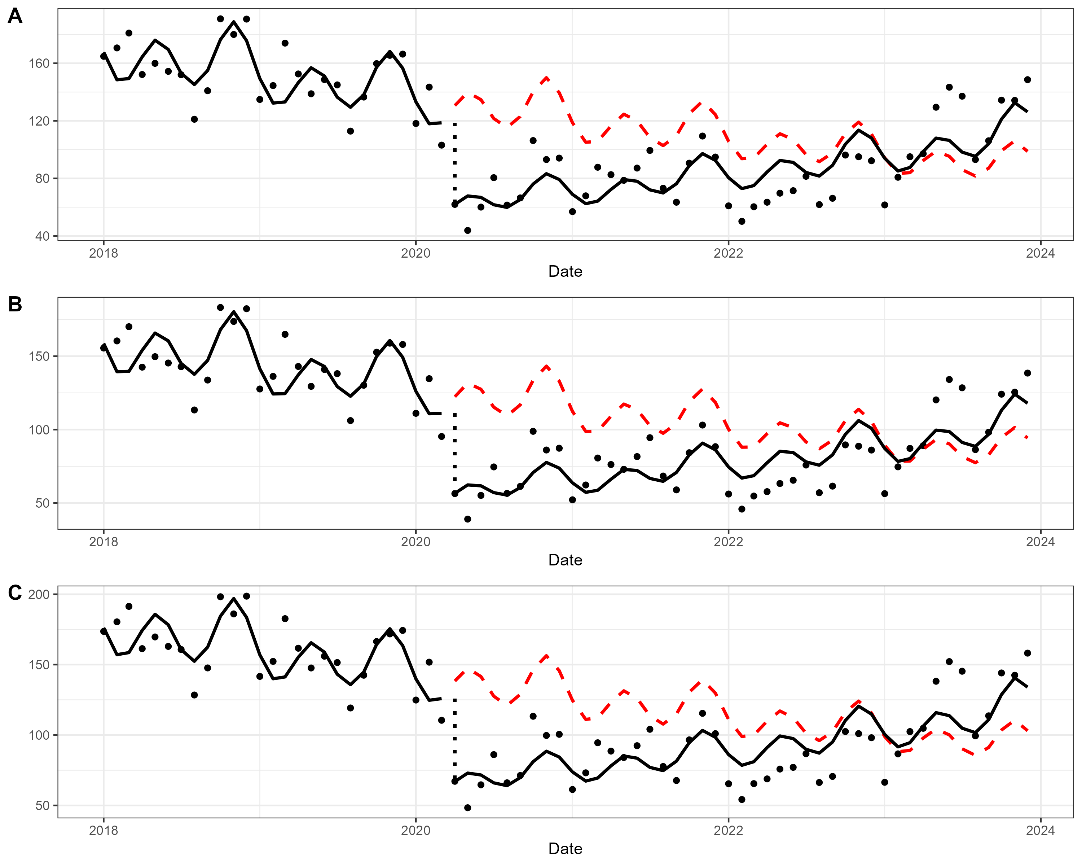** |

A: overall, B: Male children, C: Female children

**Figure S2. Broad-spectrum antibiotic prescription rate in overall and by age groups (black line: fitted values, red dotted line: predicted values had the pandemic not occurred): number of broad-spectrum antibiotic prescription fills/claims per 1000 children per month**

**Norway**

**
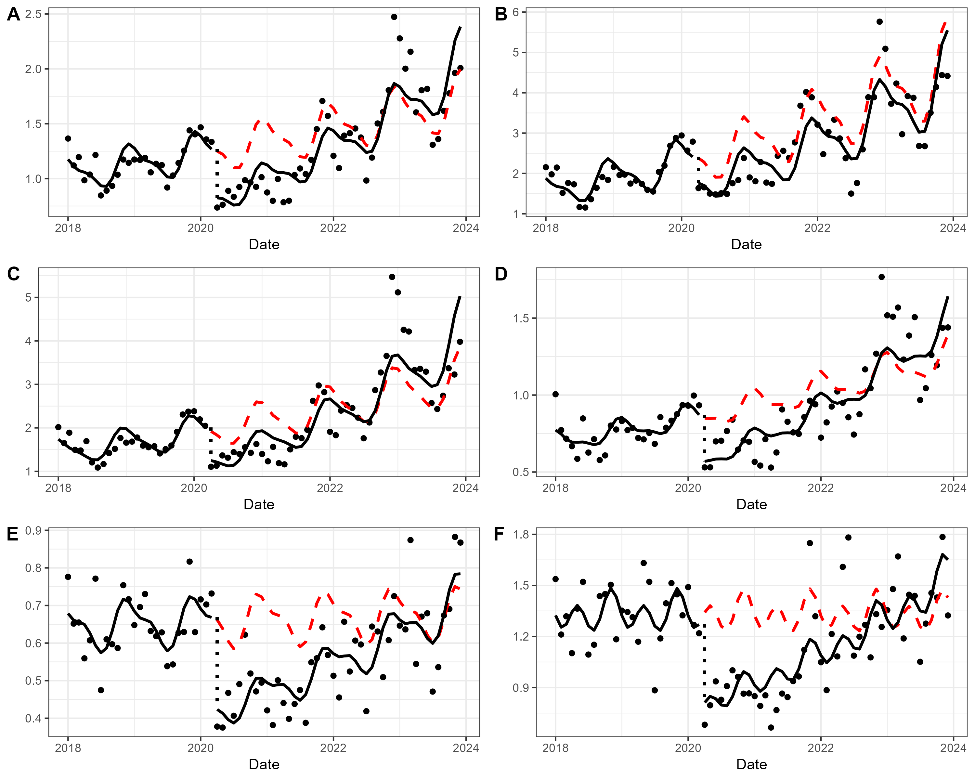
**

**Japan**

**
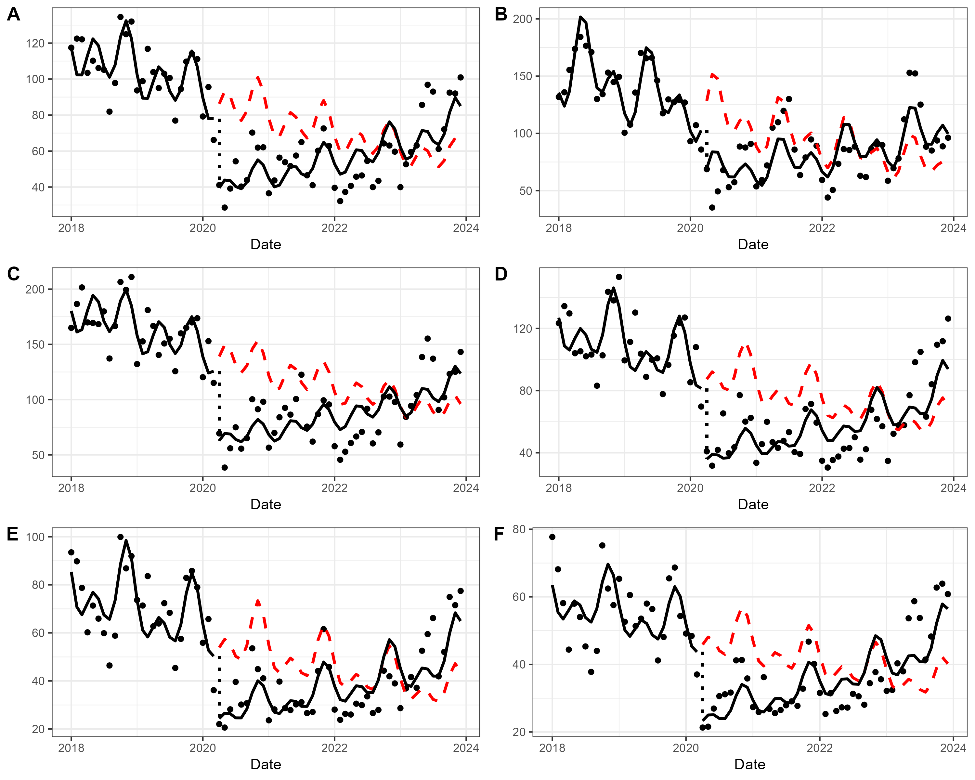
**

A: overall, B: children 0-1 years old, C: children 2-5 years old, D: children 6-9 years old, E: children 10-14 years old, F: adolescents 15-17 years old

**Figure S3. Broad-spectrum antibiotic prescription rate in overall and by sex (black line: fitted values, red dotted line: predicted values had the pandemic not occurred): number of broad-spectrum antibiotic prescription fills/claims per 1000 children per month**

**Norway**

**
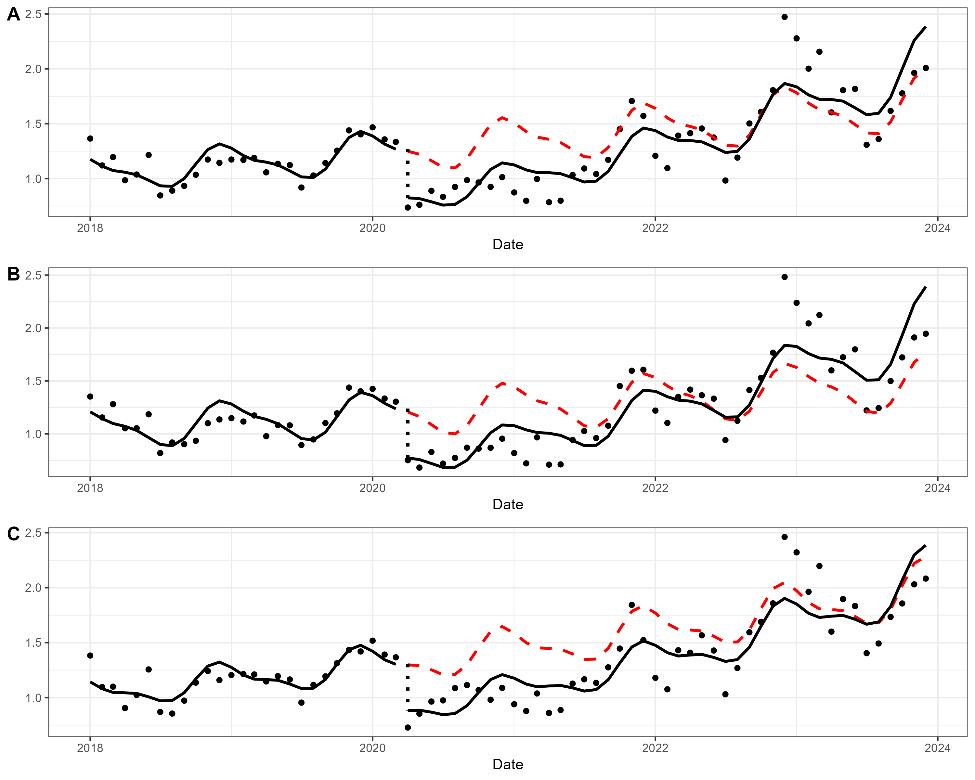
**

**Japan**

**
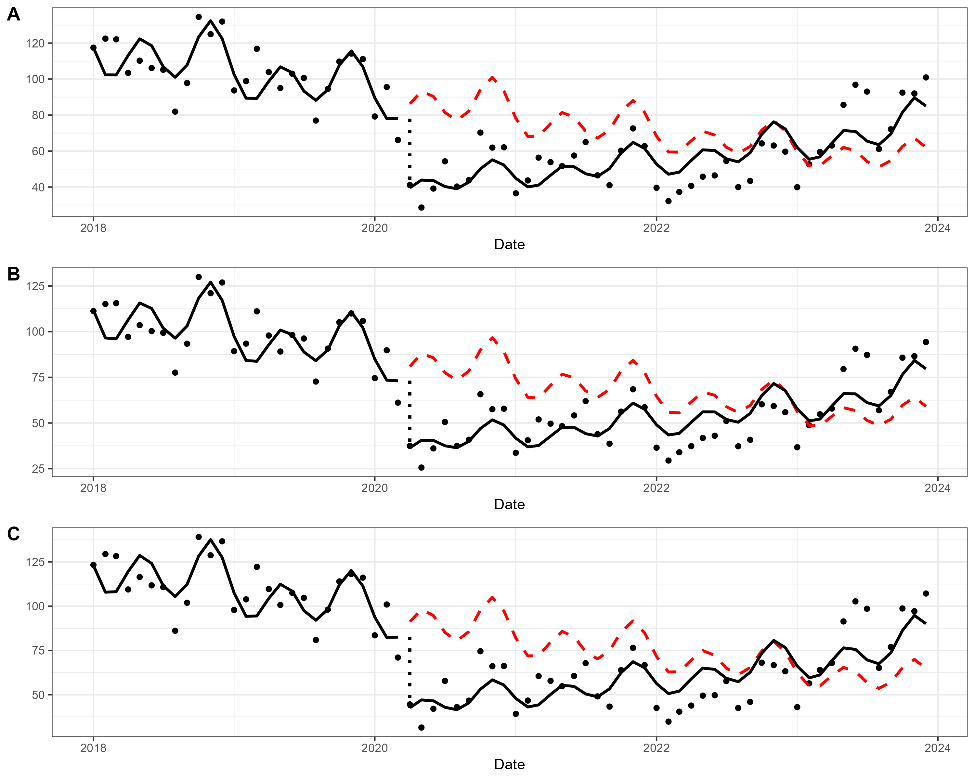
**

A: overall, B: Male children, C: Female children

**Figure S4. Proportion of antibiotic prescriptions with broad-spectrum antibiotics in overall and by sex (black line: fitted values, red dotted line: predicted values had the pandemic not occurred)**

| **Norway**  **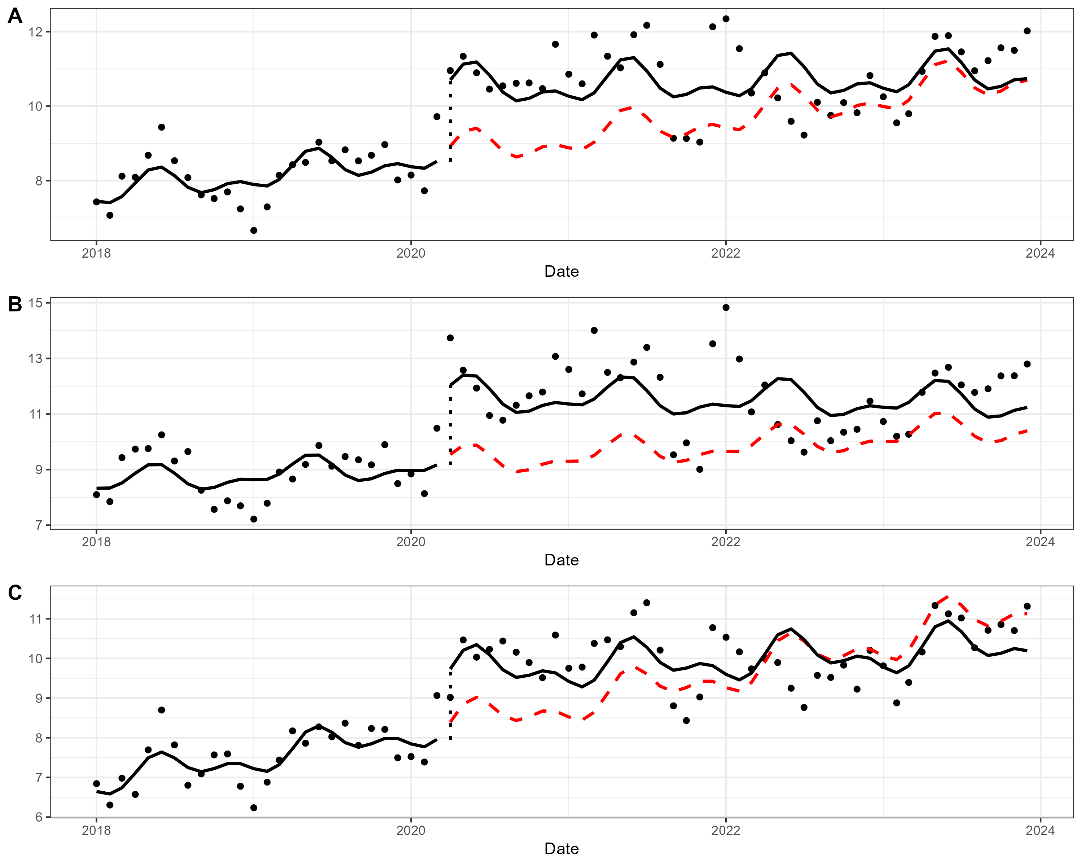** |
| --- |
| **Japan**  **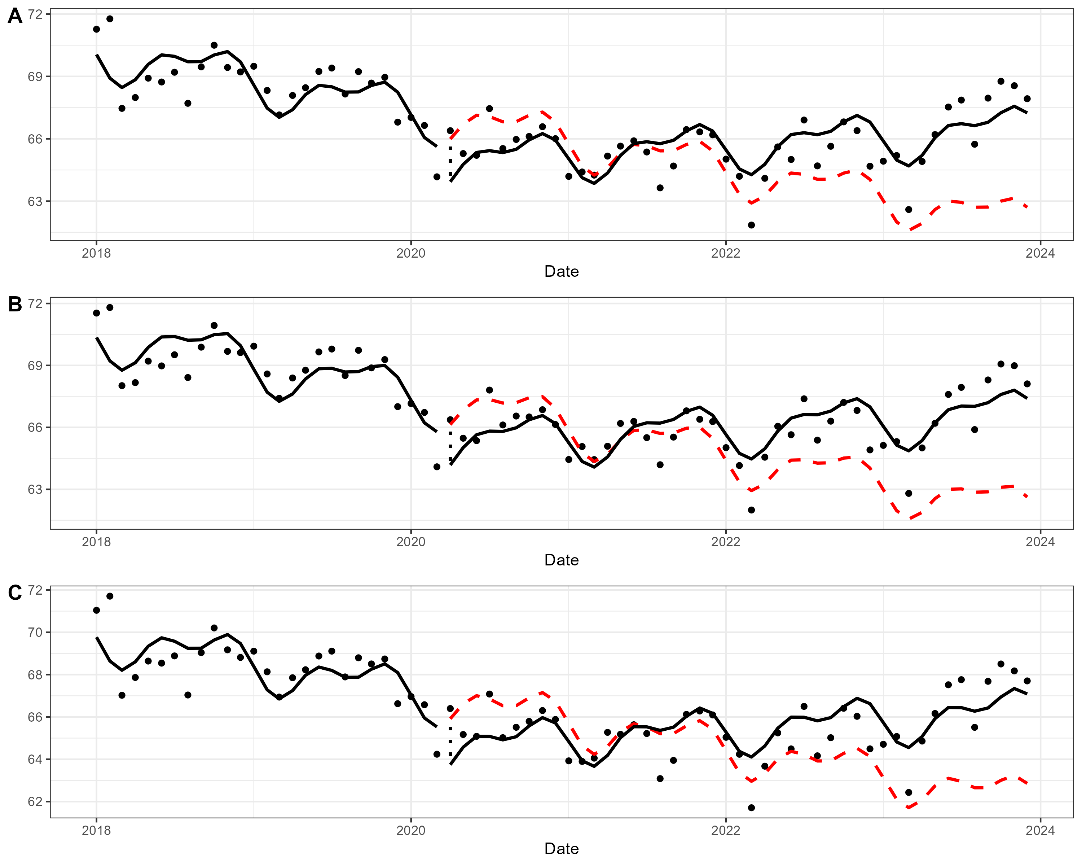** |

A: overall, B: Male children, C: Female children

**Figure S5. Proportion of antibiotic prescriptions with presumed bacterial infection diagnosis in the previous 7 days in overall and by sex (black line: fitted values, red dotted line: predicted values had the pandemic not occurred)**

| **Norway**  **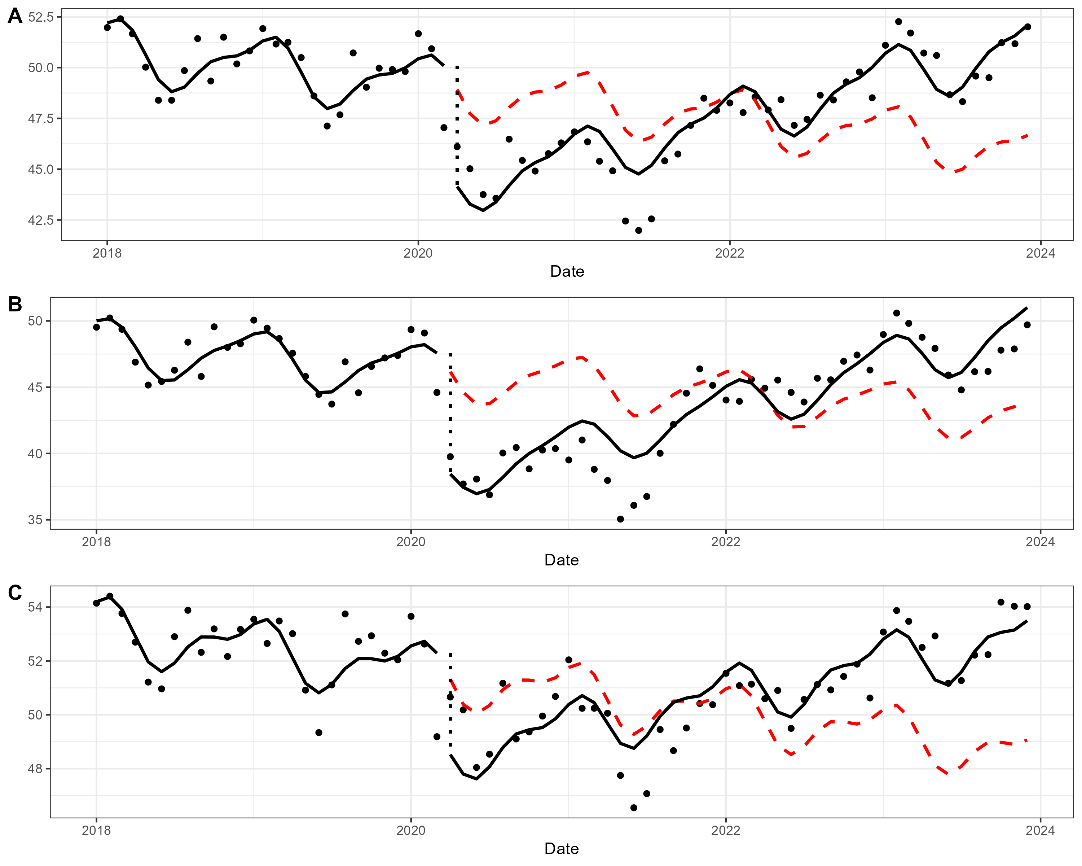** |
| --- |
| **Japan**  **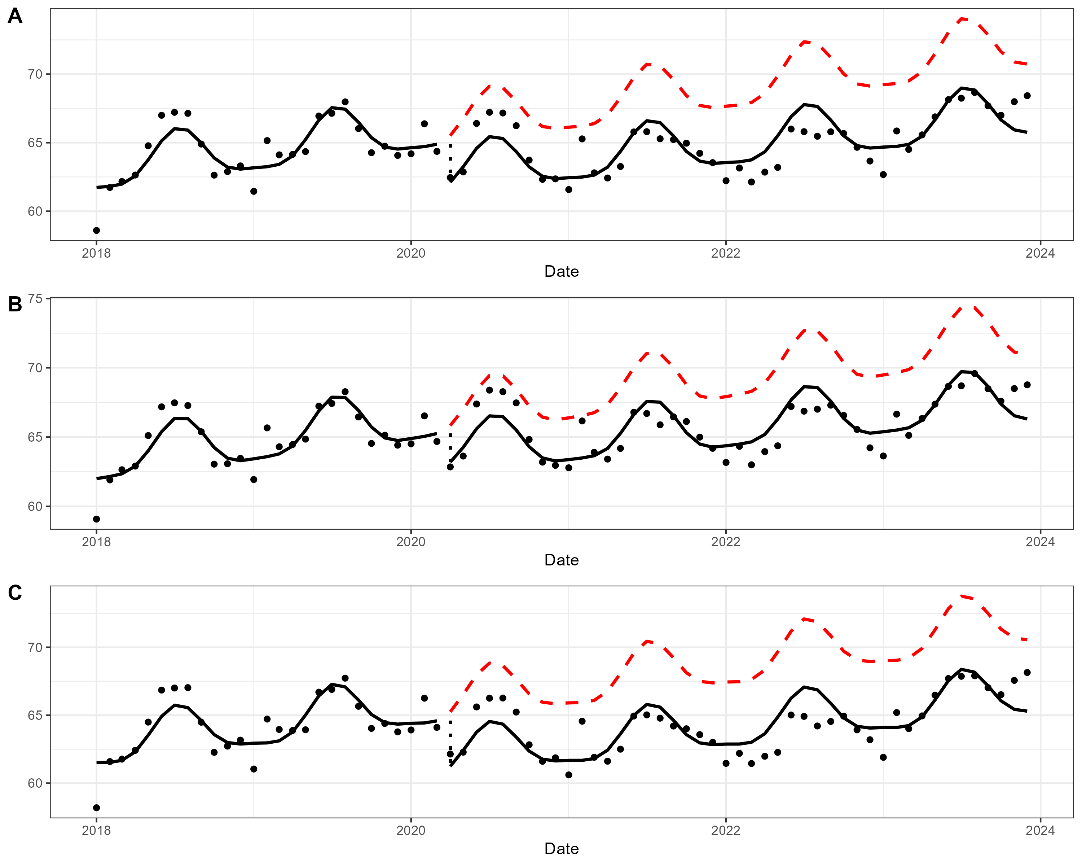** |

A: overall, B: Male children, C: Female children

**Figure S6. Rate of pediatric visit per 1000 children per month in overall and by age groups (black line: fitted values, red dotted line: predicted values had the pandemic not occurred)**

**Norway**

**
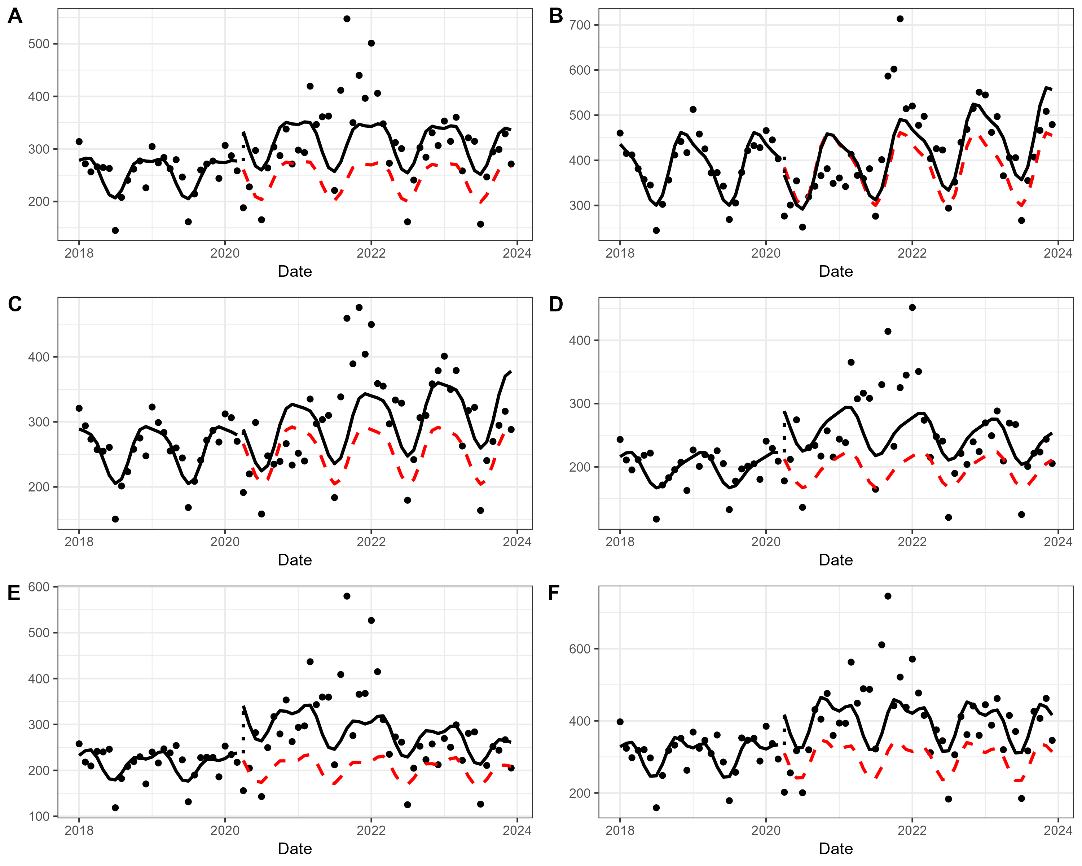
**

**Japan**

**
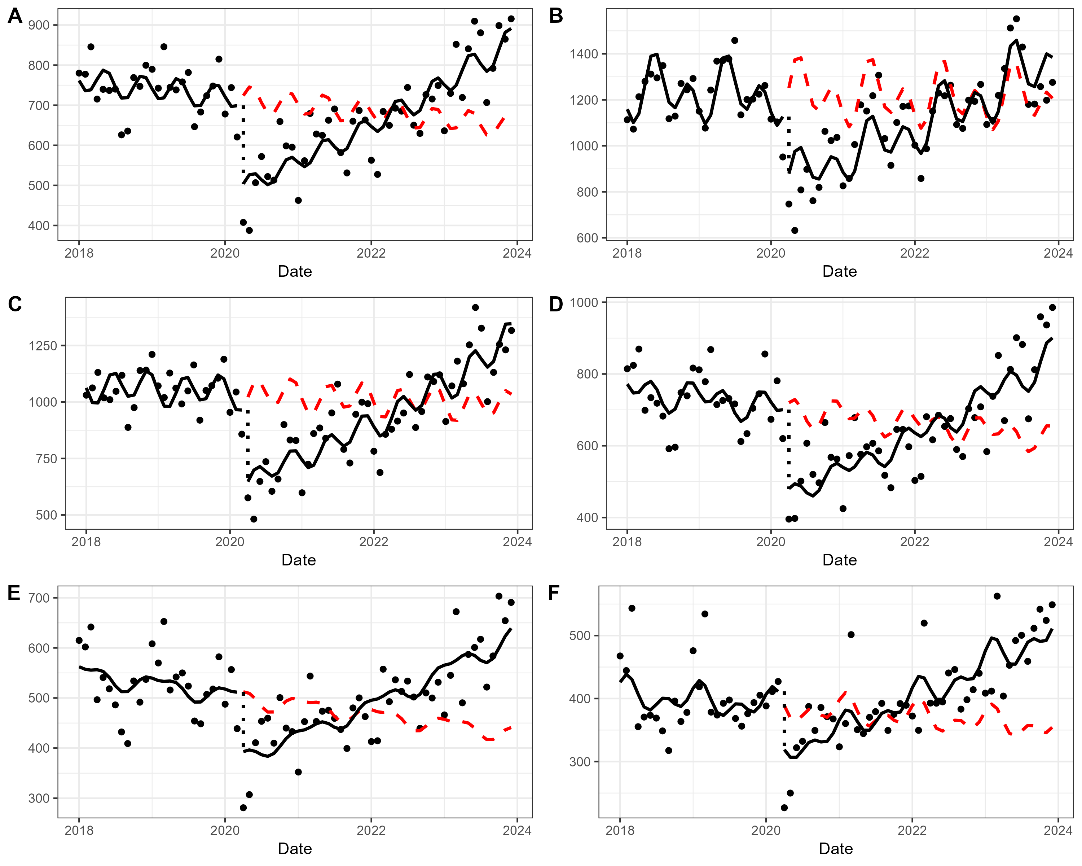
**

A: overall, B: children 0-1 years old, C: children 2-5 years old, D: children 6-9 years old, E: children 10-14 years old, F: adolescents 15-17 years old

**Figure S7. Rate of pediatric visit per 1000 children per month in overall and by sex (black line: fitted values, red dotted line: predicted values had the pandemic not occurred)**

| **Norway**  **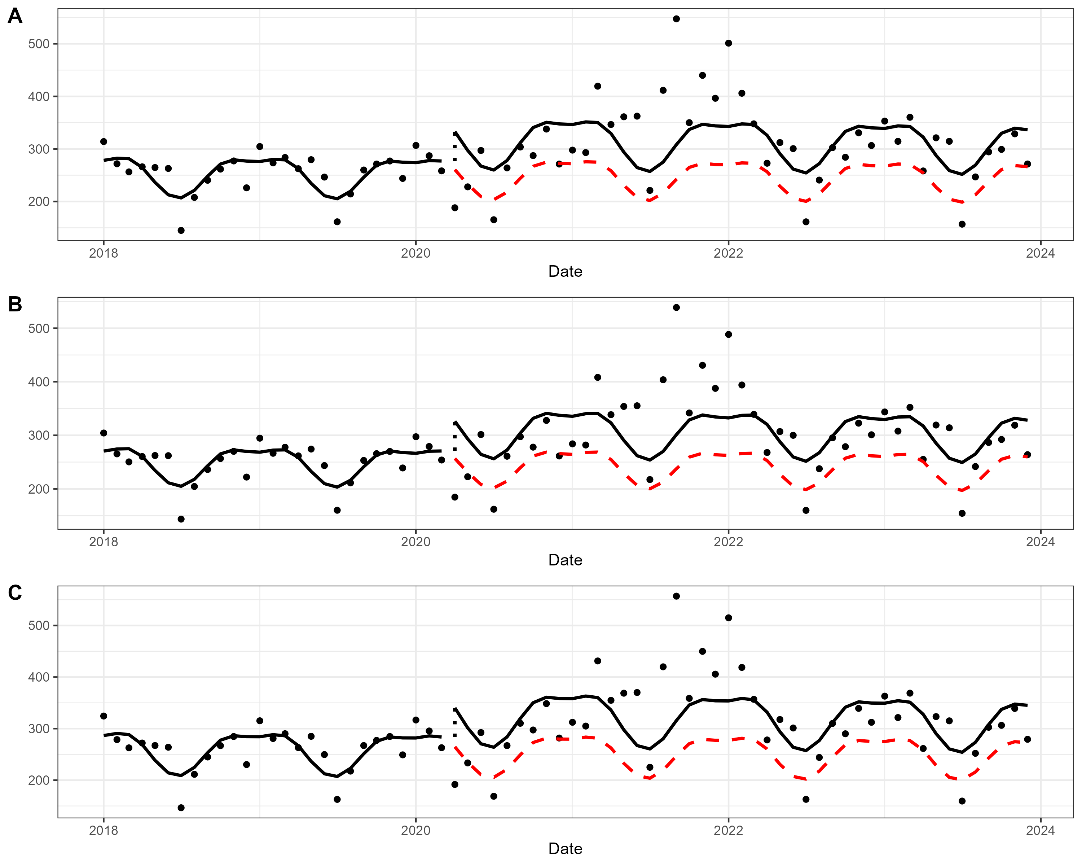** |
| --- |
| **Japan**  **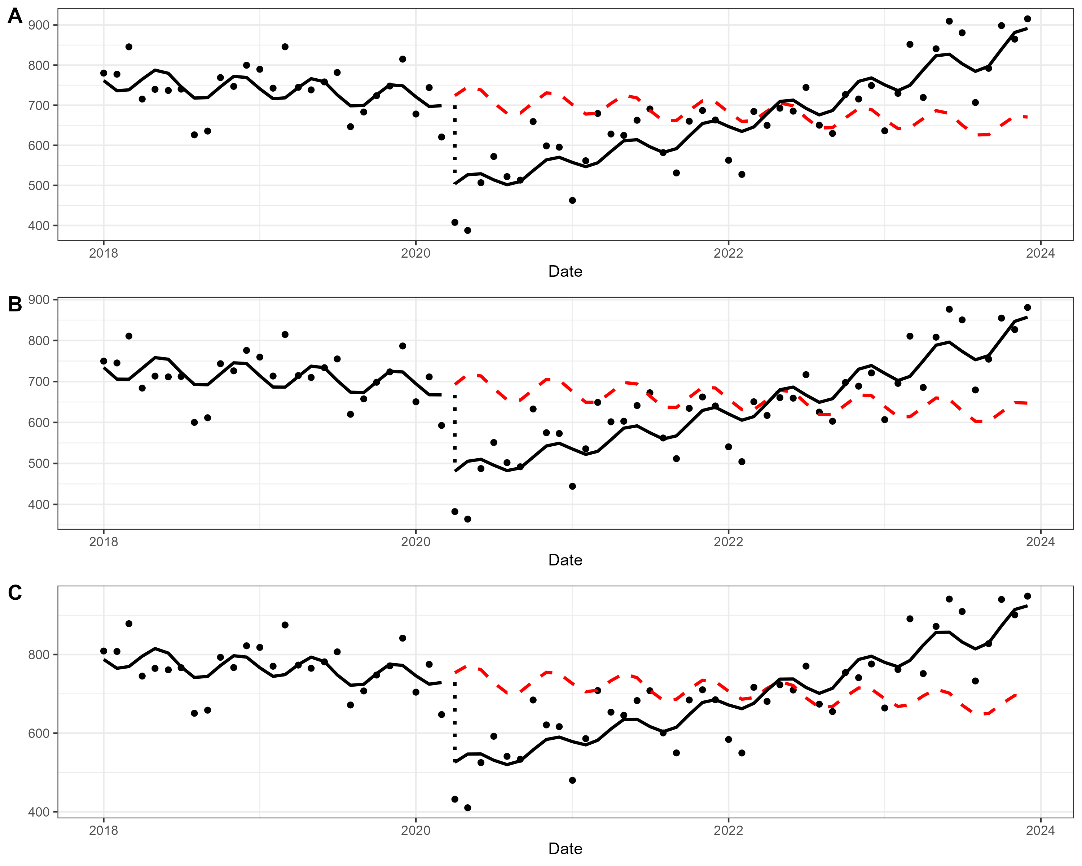** |

A: overall, B: Male children, C: Female children

**Figure S8. Rate of visits with a presumed bacterial infection diagnosis per 1000 children per month in overall and by age groups (black line: fitted values, red dotted line: predicted values had the pandemic not occurred)**

**Norway**

**
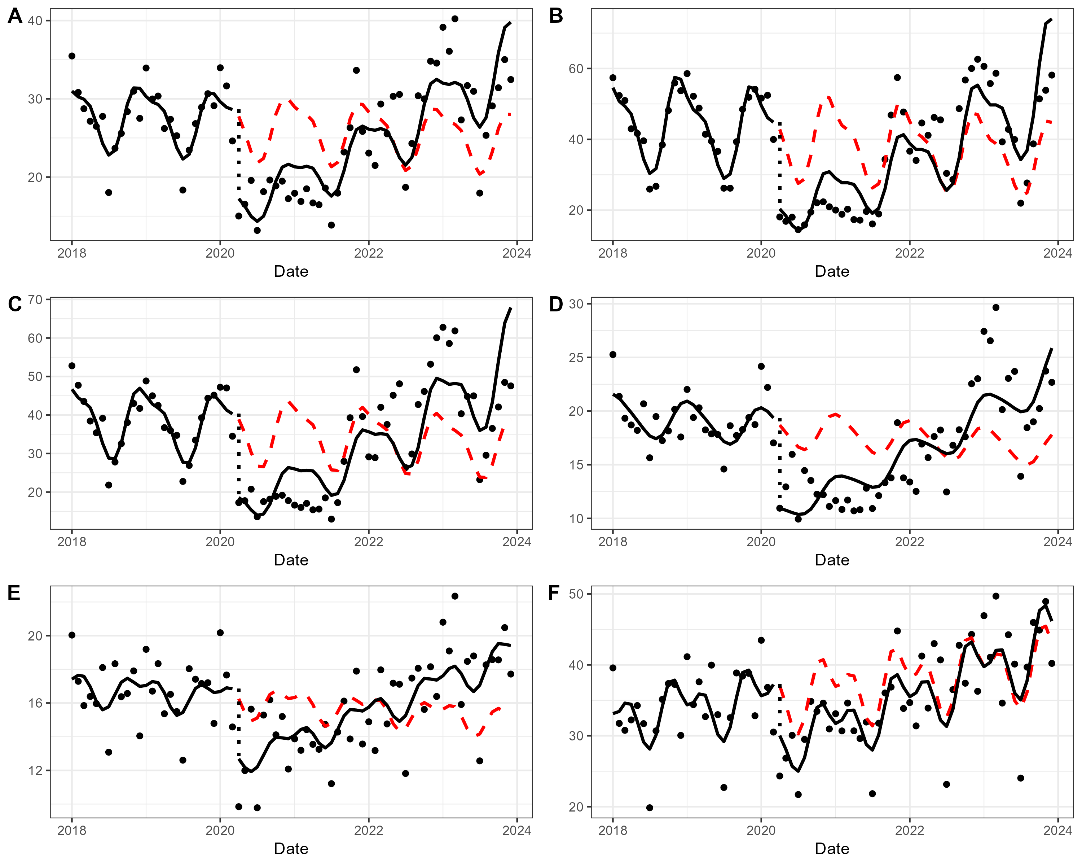
**

**Japan**

**
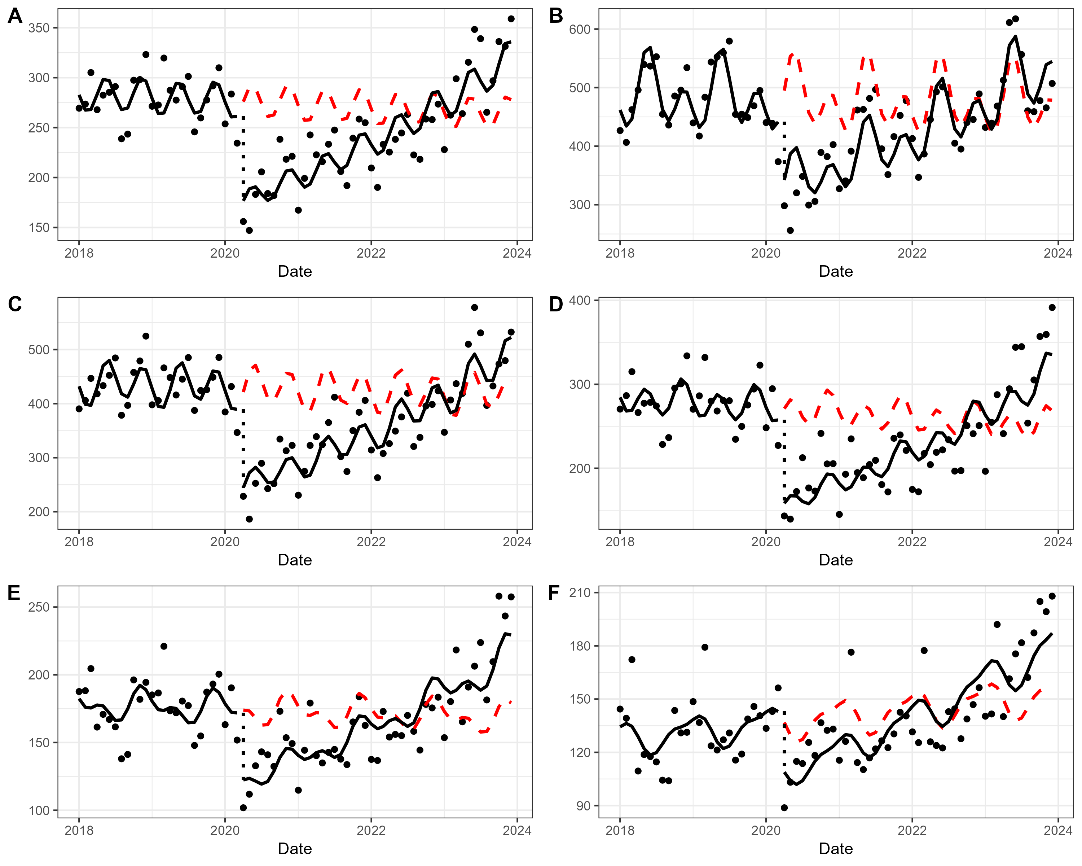
**

A: overall, B: children 0-1 years old, C: children 2-5 years old, D: children 6-9 years old, E: children 10-14 years old, F: adolescents 15-17 years old

**Figure S9. Rate of visits with a presumed bacterial infection diagnosis per 1000 children per month in overall and by sex (black line: fitted values, red dotted line: predicted values had the pandemic not occurred)**

| **Norway**  **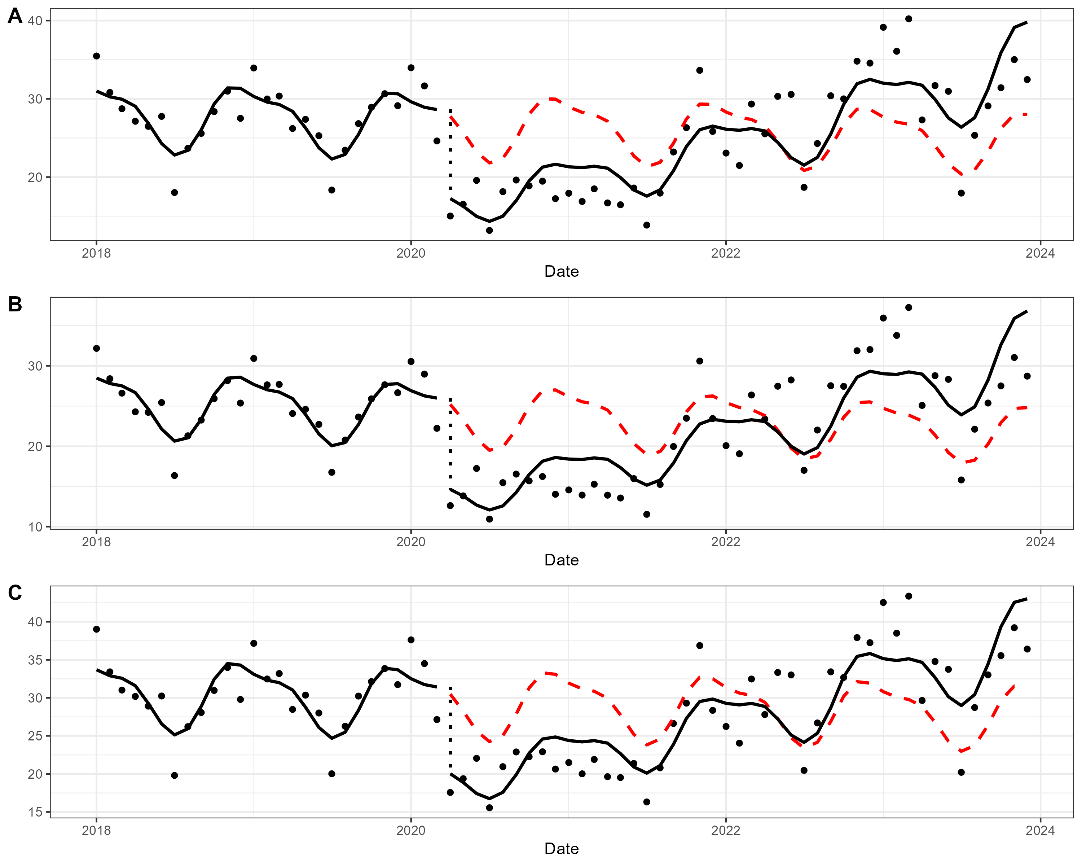** |
| --- |
| **Japan**  **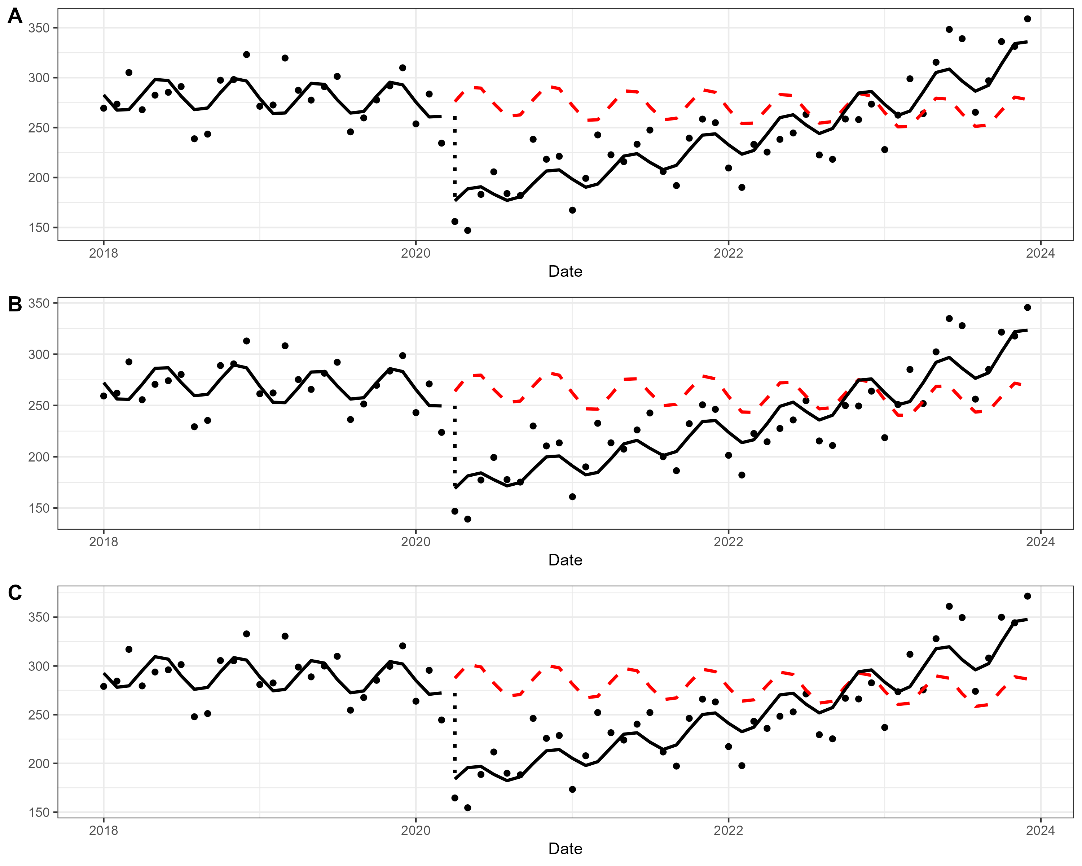** |

A: overall, B: Male children, C: Female children
